# Prevalence and Predictors of Emotional Eating among Healthy Young Saudi Women during the COVID-19 Pandemic

**DOI:** 10.3390/nu12102923

**Published:** 2020-09-24

**Authors:** Sara Al-Musharaf

**Affiliations:** 1 Department of Community Health Sciences, College of Applied Medical Sciences, King Saud University, Riyadh 11451, Saudi Arabia; salmosharruf@ksu.edu.sa; Tel.: +966-11-8050646; 2 Chair for Biomarkers of Chronic Diseases, Riyadh Biochemistry Department, College of Science, King Saud University, Riyadh 11451, Saudi Arabia

**Keywords:** Emotional Eating Scale, Increased intake, COVID-19, quarantine, stress, BMI, food consumption, young women

## Abstract

Emotional eating (EE) is prevalent among women and is associated with obesity. The coronavirus 2019 (COVID-19) pandemic and mandatory quarantine increased the risk of mental symptoms and, inferentially, emotional eating (EE). We investigated the EE prevalence and predictors during this pandemic. Overall, 638 women, ages 18–39, completed an online survey incorporating the Emotional Eating Scale, Perceived Stress Scale, Generalized Anxiety Disorder-7 Scale, Patient Health Questionnaire-9, Pittsburgh Sleep Quality Index, and Global Physical Activity Questionnaire. We asked about nutrition and collected data on weight, height, and pandemic responses. Most respondents (47.2%) reported low EE; 40.4% were “moderate” and 12.4% “high” emotional eaters; 42.8% reported depression, 27% anxiety, 71% moderate stress, and 12.5% severe stress. The main EE indicators/predictors were fat intake (β = 0.192, *p* = 0.004), number of meals (β = 0.187, *p* < 0.001), sugar consumption (β = 0.150, *p* < 0.001), body mass index (β = 0.149, *p* < 0.001), stress (β = 0.143, *p* = 0.004), energy intake (β = 0.134, *p* = 0.04), and fast food intake frequency (β = 0.111, *p* < 0.01). EE score correlated negatively with increased family income (β = −0.081, *p* = 0.049). Higher stress correlated with worse sleep, less sleep, and less physical activity. Emotional eating is common among young Saudi women during the pandemic. We recommend healthy food choices and increased physical activity to improve sleep and mitigate stress.

## 1. Introduction

Emotional eating (EE) is defined as the tendency to overeat as a coping mechanism for regulating and reducing negative emotions, such as depression, anxiety, and stress [1]. According to Bruch et al., EE represents a failure to discriminate physiologic hunger sensations from the desire to use eating as a strategy for managing negative emotions [2]. Thus, the eating behavior becomes a way to distract or escape from aversive affective states [3]. People in a negative mood state who overeat tend to consume energy-dense, palatable foods that have mood-lifting qualities typically attributed to their high sugar content [4,5], and this may lead to a corresponding weight gain [6].

The COVID-19 pandemic is a serious global health problem. By the beginning of August 2020, the World Health Organization (WHO) reported 15,581,009 confirmed cases and 635,173 deaths worldwide [7]. COVID-19 reached Saudi Arabia on 2 March 2020 [8]. Stringent public health measures, such as self-isolation and physical distancing, slowed the spread and momentarily controlled the viral outbreak in many parts of the world [9,10]. Similar to other countries, the Saudi government has sought to control infection rates by taking unprecedented measures, such as border control and formal mandatory quarantine in all cities [11]. People worked from home, schools and universities instituted distance learning, and all religious sites, including Mecca and Madinah, were locked down. While the lockdown helped to control the number of cases, at the time of the writing of this paper, there are 262,772 confirmed cases in Saudi Arabia, the highest among Gulf countries, and 2672 deaths [7].

The COVID-19 pandemic and the public health measures that have been implemented (especially mandatory quarantine) have had physical, psychological, and mental health consequences across all sectors of society [12,13]. A systematic review of 62 studies from 17 countries including China, Turkey, Iran, Spain and Italy, with a combined total of 162,639 participants, included reported rates of anxiety of 33% and depression 28% [13]. A nationwide survey in China (*n* = 52,730) reported psychological distress prevalence of 35% [12]. The negative emotional effects of this global crisis due to deaths, the mandatory quarantine, and economic disruption, along with feelings of isolation, fears of infection, stress, and disrupted lives, are all likely to increase EE [14]. Furthermore, this prolonged period of mandatory quarantine has led to psychological discomfort, sedentary behavior, disturbed sleep, and difficulty maintaining a healthy lifestyle, including making wrong food choices [15,16]. Disruptions in lifestyle, such as a sudden increase in sleep disturbances or a dramatic decrease in physical activity, are all known to trigger EE [17,18]. The overlapping of these conditions can have serious consequences because of their well-established association, either alone or in combination, with the development of short- and long-term chronic diseases such diabetes, hypertension, and obesity, as well as psychological disorders [19,20]. In the Middle East, and especially in Saudi Arabia, the prevalence of metabolic diseases and obesity is already strikingly high (39.8% and 52.9%, respectively) [21], with a higher percentage of women with obesity than men [22]. The COVID-19 pandemic and resultant quarantine restrictions may further exacerbate this endemic health crisis [23].

To date, no published studies have investigated the impact of the COVID-19 pandemic on EE, mental health, and lifestyle, especially among women of child-bearing age. Compared with men, women are more likely to develop abnormal eating patterns as well mental health disorders [19,24]; therefore, women have a higher risk of developing health problems related to eating disorders that may be precipitated or exacerbated by this pandemic.

As Saudi Arabia and other countries in the Middle East undergo epidemiologic transition and cultural change, the burden of EE is rising [25]. While anxiety, stress, and depression are central instigators of EE, few studies have been conducted to assess anxiety [26] and depression [27] in the general Middle Eastern population, and this type of mental health research is still lacking in Saudi Arabia. Therefore, the aim of this study was to assess the impact of the COVID-19 pandemic on EE in young Saudi women during the months of mandatory quarantine and to identify the main indicators/predictors of this eating disorder. Our findings could provide a framework for developing clinical and educational interventions to mitigate the short- and long-term negative impact of the pandemic on the future health of women. This study may also serve as a guide for the crafting and implementation of national public health policies with a focus on improving women’s health in Saudi Arabia.

## 2. Methods

### 2.1. Study Design and Participants

This was an observational cross-sectional study carried out among King Saud university (KSU) students and graduates in Riyadh, Saudi Arabia. A snowball sampling technique was used for this study. We created an online questionnaire using Google forms. The invitation to complete the questionnaire was sent through WhatsApp student groups and emails. Participants were encouraged to roll out the survey to as many people as possible. To provide a recruitment incentive for prospective participants, persons who completed the questionnaire received a report on their mental health and EE status. The questionnaire included eight sections, and the average time for completion was 15 min. Ethical approval for this study was obtained from the Institutional Review Board (IRB) of King Khalid University Hospital, Riyadh, before the study began (IRB number: E-19-3625). Before starting the questionnaire, each participant was required to review the aim(s) of the study and to then provide an electronic informed consent (section one). Participants could withdraw from the study at any time.

#### 2.1.1. Inclusion and Exclusion Criteria

The inclusion criteria were healthy Saudi female students (18–39 years old) or graduate participants at KSU, living in Saudi Arabia at the time the study was conducted. The questionnaire included screeners (sections two to four) that automatically excluded any participants who were non-Saudi nationals; pregnant or lactating women; and those previously diagnosed with sleep and/or psychiatric disorders, gastrointestinal disorders, significant proteinuria or amyloidosis, arthritis, anemia, malabsorption, or comorbid chronic diseases (e.g., thyroid disorders, diabetes mellitus, malignancies, and chronic obstructive pulmonary disease).

#### 2.1.2. Sample Size

Assuming an expected population standard deviation of 9.05, as obtained from the study by Schneider et al. and by employing a t-distribution to estimate sample size [28], the study required a sample size of 563 to estimate a mean with 95% confidence and a precision of 0.75. A total of 1037 participants consented to take part in the study, and 392 were excluded based on the exclusion criteria or because of incomplete and/or random responses. Recruitment and screening ultimately provided 638 participants.

### 2.2. Data Collection

Participants answered the questionnaires from 18 May 2020, to 28 May 2020. All participants included in the study completed all eight sections. In addition to COVID-19 and nutrition-related information, the participants reported general and sociodemographic information (details below). Additionally, the participants completed six standardized questionnaires that assessed their degree of EE, levels of anxiety, depression, and stress, sleep quality, and physical activity. All questionnaires had been previously validated in the Arabic language as referenced below.

### 2.3. Variables Measured

#### 2.3.1. General and Sociodemographic Information 

These variables included contact data (email or mobile phone number), age, date, city of birth, and smoking (yes/no). Other variables were affiliated university department, education level, grade point average (GPA), employment status, income, marital status, parental status, and number of children, and area of residence.

#### 2.3.2. COVID-19- Related Knowledge 

These questions collected information about the following: infection by Covid-19 (self or relatives), type of quarantine (home/mandatory institution) [16], and income change due to COVID-19 (increase/decrease/no change) [29], food cleaning (yes/no), and change of residency

#### 2.3.3. Nutrition- Related Information

Participants were asked to report their height in cm and their weight in kg and these values were used to determine the body mass index (BMI, kg/m^2^). The World Health Organizations (WHO) categorizes BMI cutoffs into four groups: underweight (<18.5 kg/m^2^), normal weight (18.5–24.9 kg/m^2^), overweight (25.0–29.9 kg/m^2^), and obese (≥30 kg/m^2^) [30]. Questions related to the mandatory quarantine period included weight change because of lockdown (increase/decrease/no change), following a weight loss diet (yes/no), number of meals and snacks per day, fast food intake and its frequency, and the frequency of eating or the urge to eat sweets (Likert-type scale [hereafter “Likert scale”]), urge to drink coffee and tea (Likert scale), and amount of water intake. Additionally, food frequency questionnaire was conducted to assess macronutrients intake including energy intake (kcal/day), fat intake (gm/day), carbohydrate intake (gm/day) and protein intake (gm/day) [31].

#### 2.3.4. Emotional Eating Scale (EES)

This study used a validated Arabic version of the Emotional Eating Scale (EES) [32]. The EES is composed of twenty-five self-reported items that assess the urge to eat under the influence of negative emotions, including anger, anxiety, and low mood state (depression) [33]. Participants rate their answers using a five-point Likert scale ranging from 0 (no desire to eat) to 4 (an overwhelming urge to eat). The total score is calculated by summing the scores of all the items, and can range from 0–100, with the higher scores indicating a reliance on using food to help manage emotions. The EES has been established in both clinical [33] and non-clinical samples [34]. The EES had a good test-retest reliability (*r* = 0.79, *p* < 0.001) and internal consistency Cronbach’s alpha was 0.81 [32]. No cutoff is available for EES; hence, z-scores were used to produce three groups. Values greater than mean + sd were treated as high while mean–sd were treated as low, while values between these two extremes were treated as moderate values, to assess the prevalence. The following cutoffs were produced: scores ≤27.5 indicated low EE, scores between 27.6 and 43.6 indicated moderate EE, and scores ≥43.7 indicated high EE. A composite score for each subscale was calculated by summing the total of the endorsed items that corresponded to each EE component. In this study, the Cronbach’s alpha was 0.8.

#### 2.3.5. Perceived Stress Scale (PSS) Questionnaire

The Perceived Stress Scale (PSS) Questionnaire is a commonly used 10-item instrument developed to quantify perceived stress [35]. This study used the validated Arabic version [36] to assess how participants perceived stress due to the pandemic and the quarantine. The questions concerned specific stressors or thoughts about stressful events taking place during the past month, potentially addressing both chronic and acute effects of, and responses to, stressful events and activities. Each item was answered using a 5-point Likert scale, with scores ranging from 0 (almost never) to 4 (almost always), where higher scores indicated a more severe perceived stress [37]. Summed scores ranged from 0 to 40. Stress was stratified into three groups: high stress (score ≥ 27), moderate stress (14–26), and low stress (≤13) [37].

#### 2.3.6. Generalized Anxiety Disorder-7 (GAD-7)

We used the Arabic version of the Generalized Anxiety Disorder-7 (GAD-7) scale to assess symptoms of anxiety [38]. This scale has been validated and used in the Arabian population [38,39]. Seven items measured the frequency of anxiety symptoms over the past two weeks on a 4-point Likert scale ranging from 0 (never) to 3 (almost every day). The GAD-7 total score ranged from 0 to 21, with increasing scores indicating a greater severity of functional impairments as a result of anxiety [40]. A cutoff score of 10 or more was used as a positive screen for the presence of generalized anxiety disorder, as this was the most commonly used cutoff and the one that best balanced sensitivity and specificity [40].

#### 2.3.7. Depressive Symptoms

The Patient Health Questionnaire-9 (PHQ-9) is a subscale of the PHQ questionnaire designed to assess symptoms of depression [41]. This study used the Arabic version that has been validated and extensively used in the Arabian population [38,39]. Nine items measure the frequency of depression symptoms over the past week on a 4-point Likert scale ranging from 0 (never) to 3 (nearly every day). Total scores range from 0–27, with higher scores indicating greater depression [41]. In this study, we used a cutoff of 10 and above as a positive screen for depression [39,41].

#### 2.3.8. Sleep Quality 

We used the Arabic language Pittsburgh Sleep Quality Index (PSQI) [42]. Seven components (subjective sleep quality, sleep duration, sleep latency, habitual sleep efficiency, use of sleep medications, sleep disturbance, and daytime dysfunction) were scored from 0 to 3 points. The global PSQI score ranges from 0 to 21, with higher scores indicating a more severe sleep disorder [43]. A score ≥5 indicates poor sleep quality [43].

#### 2.3.9. Physical Activity

This study applied the Arabic version of the Global Physical Activity Questionnaire (GPAQ) version 2.0 [44], which was also previously used in a college-age Saudi population [45]. The questionnaire covers several components of physical activity, such as intensity, duration, and frequency. It also assesses three domains in which physical activity was performed: occupational physical activity, transport-related physical activity, and physical activity during discretionary or leisure time. Participants were also asked to record their daily steps by recalling a number of average weekdays.

### 2.4. Statistical Analyses

Data were analyzed using SPSS version 22.0. Continuous variables were presented as the mean ±SD, while categorical variables were presented as *n* (%). Differences in means and percentages were calculated using independent sample t-test, ANOVA, and chi-square test of independence (used in supplementary tables). Correlations between continuous variables were obtained with the Pearson correlation test. Linear regression was used to identify indicators/predictors of emotional eating. Assumptions of linearity were verified, and multicollinearity was checked using the variance inflation factor (VIF) with cutoff of 10. All VIF values were way below than 10 indicating no multicollinearity existed. A *p*-value < 0.05 was considered statistically significant.

## 3. Results

### 3.1. General Characteristics

A total of 638 young women, with a mean age of 22.0 years ± 1.9 years, were studied. The mean BMI during quarantine was 23.2 ± 5.0 kg/m^2^, with 173 (27%) women categorized as obese and overweight (≥25 kg/m^2^). The participants’ sociodemographic characteristics are presented in Table 1. More than half of the women (413; 60%) were undergraduates, and the majority of the sample was unemployed (579; 91%). The family income for 254 (40%) women was >20,000 Saudi Riyals/month. The majority of the women were single (442; 95%), 22 (4.7%) were married, and 12 (3.5%) had children. Of the total, 259 women (41%) lived north of Riyadh.

### 3.2. Baseline Characteristics

Approximately half of the women sampled reported EE. The mean total EES score was 27.5 ± 16, the EE-depression subscale score was 10.5 ± 6.6, the EE-anxiety subscale score was 4.4 ± 2.5 and the EE-anger subscale score was 5 ± 4. In total, 335 women (52.5%) reported low EE, while 202 (31.7%) were in the moderate and 101 (15.8%) were in the high EE groups (Table S1). Women in the moderate EE group (21.7 ± 1.7) were younger than women in the low and high EE groups (22.1 ± 1.9 and 22.3 ± 2.3, respectively; *p* = 0.006). As might be expected, a greater number of women reported being obese or overweight in the high (16.8% obese, 23.8% overweight) than in the low (6.3%, 16.8%, respectively) and moderate (9.9%, 17.3%, respectively) EE groups (*p* = 0.003). BMI was significantly greater in the high than in the low and moderate EE groups (25.4 ± 6.3 vs. 22.7 ± 4.9 vs. 23.4 ± 6.4, respectively) (*p* < 0.001). No significant differences were noted in education or residence between the groups (Table S1).

The prevalence of high EE appeared to be inversely related to family income (Table S1). The two highest tiers of family income (>20,000 SAR and 10,000–20,000 SAR) were associated with the highest reports of high EE (43.0% and 41.8% respectively). For both the lowest (<5000 SAR) and next lowest (5000–10,000 SAR) income tiers, high EE was low (each 7.6%). EE was generally reported as low across levels of severity in the lowest income tier. In the next lowest tier, low and moderate EE roughly split the difference in terms of reported prevalence between adjacent tiers. Note that the sample skewed toward higher income, with the number of participants roughly doubling, from lower to higher through the lower three tiers to rough numerical parity in the top two, making extrapolation hazardous. The patterns, while suggestive, did not reach statistical significance (Table S1).

### 3.3. Mental Status Parameters

The stress, depression, and anxiety mean scores were 19.2 ± 6.2, 9.3 ± 5.4, and 7.2 ± 4.7, respectively. A total of 42.8% of the women reported being depressed and 27% were anxious; 71% reported moderate stress and 12.5% reported severe stress (Table 2).

### 3.4. COVID-19 Pandemic-Related Parameters

During the pandemic, 84% of women did not experience any change in income, and 93% did not change residence. Only one woman (0.2%) was infected with COVID-19, and 12 (2%) reported either their parents (30%) or other family members (70%) had been infected. The overwhelming majority (99.7%) of women were under mandatory quarantine. Two women (0.3%) were hospitalized during the quarantine. There were no significant differences in any of these parameters between the EE groups (Table S2).

### 3.5. Correlations for EES, PSS, PHQ-9, and GAD-7 Scores

The total EES score was positively correlated with BMI (*r* = 0.11, *p* < 0.005), the GPAQ (*r* = 0.02, *p* < 0.05), and the total PSS scores (*r* = 0.13, *p* < 0.005). The total EES score was also positively correlated with the number of meals per day (*r* = 0.2, *p* < 0.005), energy intake (*r* = 0.13, *p* < 0.05), fat intake (*r* = 0.2, *p* < 0.005), and protein intake (*r* = 0.15, *p* < 0.05). The EE-anxiety score was positively correlated with the PHQ-9 (*r* = 0.09, *p* < 0.05) and GAD-7 (*r* = 0.12, *p* < 0.005) scores (Table 3).

The PHQ-9 depression subscale score was positively correlated with the GAD-7 (*r* = 0.64, *p* < 0.001) and PSS (*r* = 0.521, *p* < 0.001) scores. The PHQ-9 score was positively correlated with the global PSQI score (*r* = 0.25, *p* < 0.001) and negatively correlated with the duration of sleep (h/day) (*r* = −0.18, *p* < 0.001). More depression was positively correlated with more sitting time (*r* = 0.086, *p* < 0.05) and negatively correlated with daily steps (*r* = −0.15, *p* < 0.001). Women with higher depression scores ate fewer meals per day (*r* = −0.19, *p* < 0.005) (Table 3).

The GAD-7 anxiety score was positively correlated with a higher global PSQI score (worse sleep) (*r* = 0.178, *p* < 0.001) and negatively correlated with duration of sleep (hours/day) (*r* = −0.21, *p* < 0.001). The GAD-7 was also positively correlated with less activity, indicated by longer sitting time (*r* = 0.107, *p* < 0.001). Lastly, a high anxiety score was negatively correlated with the number of meals (*r* = −0.139, *p* < 0.001) while correlating positively with higher fat intake (g/day) (*r* = 0.143, *p* < 0.05) (Table 3).

The PSS stress score was positively correlated with both anxiety and depression scores (*r* = 0.521, 0.528, *p* < 0.001), respectively. The stress score was positively correlated a higher global PSQI (worse sleep) (*r* = 0.159, *p* < 0.001) and negatively correlated with duration of sleep (h/day) (*r* = −0.196, *p* < 0.001). A high stress score was positively correlated with less activity, indicated by longer sitting time (*r* = 0.08, *p* < 0.05) and negatively correlated with steps per day (*r* = −0.224, *p* < 0.001). Furthermore, a high stress score was positively correlated with the number of snacks (*r* = 0.084, *p* < 0.05) and negatively correlated with the number of meals (*r* = −0.01, *p* < 0.05) (Table 3).

### 3.6. Indicators and Predictors of EES Score

Multiple linear regression analyses revealed fat intake (β = 0.192, *p* = 0.004), number of meals (β = 0.187, *p* < 0.001), sugary food consumption (β = 0.150, *p* < 0.001), BMI (β = 0.149, *p* < 0.001), stress (β = 0.143, *p* = 0.004), energy intake (β = 0.134, *p* = 0.043), and fast food intake (β = 0.127, *p* = 0.005) were positively correlated with the EES. An increase in family income (β = −0.081, *p* = 0.049) was negatively associated with the EES score. The standardized beta demonstrated the relative importance of each predictor of the EES. Thus, fat intake was the most important predictor of EES score, followed by the number of meals, sugary food consumption, BMI, the PSS score, energy, and fast food intake, with an increase in family income being a negative predictor. Further analysis revealed that a change of one standard deviation in nine parameters yielded the following respective changes in standard deviation in the EES: fat intake 0.178, number of meals 0.173, sugary food consumption 0.155, BMI 0.153, eating fast food at least 2–4 times a week 0.136, PSS score 0.130, energy intake 0.130, eating fast food 1–4 times a month 0.114, and an increase in family income −0.08 (Table 4). All multiple linear regression models yielded an adjusted R^2^ value below 10%.

## 4. Discussion

To the best of our knowledge, this is the first study to assess EE among young Saudi women, as well as its predictors during the COVID-19 pandemic. We found that EE is very common among young women during this pandemic, with almost one in two women identifying as emotional eaters. Of these, 12.4% were in the high EE group. An Italian study during COVID-19 addressed emotional eating using a subset of items from the Yale food addiction scale. Among 602 individuals (age 18–79 years old), about half the cohort increased their food intake to feel better (55%) and used food in response to anxious feelings (48.7%) [46]. The study identified women as being more disposed than men to anxiety and to consuming comfort foods. Women were also more likely to report depression; EE (and specific anxiety about it); treatment with drugs and supplements; and having been “on a diet” prior to COVID-19. Younger age, lower BMI (significant for women respondents compared to men), not being anxious, and less food intake for satisfaction were predictors of overfeeding control [46].

Comparable studies of disordered eating in the Middle East are scarce and were all carried out before the COVID-19 pandemic. They provide some limited insight into the prevalence of eating disorders in the Middle East before the pandemic, albeit in different populations. Most studies assessed binge eating [47] among the obese population [48] rather than EE. In 2016, Schulte et al. reported a median EES score of 23.5 among 236 young men and women in the United Arab emirates [49]; Annesi and Mareno reported similar pre-pandemic EES results in 2015 in the United States at 25.5 ± 11.3 in a population of women 21 years and older with obesity [50]. Our higher mean total EES score of 27.5 ± 16 suggests the likelihood of a COVID-19 effect on emotional eating. Other studies on EE differed from our investigation in a number of substantial ways, making a direct comparison problematic. For example, some lacked a standard definition of EE, used a different EE tool, did not report the total EES score, or included a much broader age range of subjects [50,51,52].

The percentage of our respondents who reported being anxious (27%), depressed (42%), or moderately (71%) or severely stressed (12.5%) is consistent with findings from studies carried out in Saudi Arabia, Spain, India, and China that evaluated mental health issues during the COVID-19 pandemic [29,53,54,55]. In our study, a trend was evident toward higher-scoring emotional eaters reporting more anxiety and depression. Anxiety, depression, and stress scores were also highly correlated with each other and were individually associated with worse sleep quality, shorter sleep duration, less physical activity, and more sitting time. Women who were anxious, depressed, and stressed ate fewer meals per day, while women who were stressed ate more snacks per day. No correlation was found between the global sleep PSQI score and physical activity and the EES score.

### 4.1. Indicators and Predictors of EE

#### 4.1.1. Type and Quantity of Food Consumption

Not surprisingly, the strongest indicators of EE among young women were fat intake, number of meals, sugary food consumption, and more frequent fast food intake. This finding is consistent with the mood-lifting effects associated with this type of consumption of palatable fatty and sugary meals [4,5]. Physiologically, consuming fat and sugary food increases the production of serotonin and dopamine, positively affecting mood [56]. We posit that, during the mandatory quarantine and consistent with a previous report [5], EE evolves as an adaptive mechanism for managing negative emotions, which in turn serve as a psychological determinant of EE [5]. Our results are further consistent with those of van Strien et al. [57], who showed that, after the induction of a negative or positive emotional state, high but not low emotional eaters increased their food intake. A recent systematic review of experimental studies also found that participants in whom negative emotions had been induced tended to score higher on the EES and eat more energy-dense food compared to controls who experienced neutral emotions [4]. Both studies proposed that participants used their eating behavior as a coping or healing strategy to manage their emotional state [4,57].

However, not all studies have reached similar conclusions. For example, Evers et al. found that negative feelings were not associated with overeating in overweight or obese individuals, in individuals with eating disorders, or in self-assessed emotional eaters [58]. This discrepancy may, in part, be due to the use of different cutoff points for classifying EE, to variations in the emotion induction procedures, and/or to differences in sociodemographic characteristics of the population studied.

#### 4.1.2. Obesity

After consumption patterns, a signal predictor for EE among young women was obesity. Obesity is considered a biological determinant of EE. In a recent US study conducted during COVID-19 (*n* = 123), respondents who reported weight gains of 2.3–4.5 kg (22% of sample) also reported that they lacked sleep, were less physically active, snacked more after dinner, ate more in response to stress, and more often ate because of the sight and smell of food, when compared to respondents who did not change these behaviors at all [59]. The researchers, however, did not report if this behavior was associated with BMI. Previous studies have shown an association between EE and BMI [60,61]. Individuals who were overweight and experienced negative emotions were found to eat more than normal-weight and underweight individuals [61]. Another study involving 1453 students at a public university in Mexico City showed that emotional eating correlated with BMI in both men (β = −0.08, *p* < 0.001) and women (β = −0.09, *p* < 0.001) [60]. People who are obese may already have a prolonged history of engaging in EE and have consequently gained more weight over time. Additionally, the level of dietary restraint may be predictive of emotional eating among individuals who are obese [62]. A noteworthy observation from our study was that women who were dieting reported more emotional eating; while this trend did not reach statistical significance, it is consistent with the findings of the previous studies.

#### 4.1.3. Sex

Consideration of potential biological determinants of EE must include female sex—which by the nature of our all-female cohort, may partly explain the high percentage of EE in this study. Interestingly, studies have shown that women, as opposed to men, experience more EE by eating more and making palatable food choices [63]—behavior that has been linked to mood swings caused by hormonal changes related to the menstrual cycle [64].

#### 4.1.4. Stress

Stress was the next most important predictor of EE among women in the current study, and is, of course, expected under the circumstances of pandemic and quarantine. Stress and its behavioral correlates make up the next block of EE associations we found. Consistent with previous studies stress was shown to prompt overeating, especially among women [49,65,66]. We found that higher stress scores were positively correlated with a high number of snacks and fast food consumption. Similar to our findings, a recent study conducted before the COVID-19 pandemic among 360 respondents via Amazon Mechanical Turk that experienced post-traumatic stress, showed that the severity of post-traumatic stress symptoms predicted greater difficulties in regulating emotions, which also led to more EE [66]. In an experimental study by Mantau et al. of 179 participants from a university environment (mean age 23.15 years), participants’ mood states were manipulated, and their food choices were assessed. Only stressful situations were significantly associated with EE and led to more than 70% of the unhealthy food choices [67]. By contrast, another study [68] found no association between stress and EE among 169 undergraduate students, nor did it find any association between EE and fear, anger, sadness, or depression. These disparate results may be explained by the small sample sizes, the use of different instruments to measure EE and stress, and variations in the level of stress induced.

Numerous psychological and physiological hypotheses have been proposed to explain the association between stress and EE. Emotional eaters may perceive the negative feelings of stress as hunger, for which eating is the obvious solution [69]. Suppression of emotions or maladaptive coping strategies, such as a reliance on emotion-oriented coping and avoidance of stress by distraction, have also shown positive associations with EE [70]. Chronic stress, like that experienced by women during the COVID-19 pandemic, also leads to a number of physiological changes, such as increases in cortisol secretion, which can, in turn, stimulate hunger sensations [71]. Another proposal is that chronic stress may lead to a paradoxical *hypo*-activation of the hypothalamic pituitary axis (HPA) and atypical neurovegetative symptoms of increased appetite and EE [72,73].

#### 4.1.5. Anxiety

This study did not find any significant association between anxiety and EE. One potential explanation is that anxiety, similar to *acute* stress, is an acute feeling of high-intensity or high-arousal emotions, such as fear, and tends to suppress eating as these are related to physiological and behavioral responses that reduce appetite and interfere with eating [74]. In accordance with this observation, our study showed that women with anxiety ate fewer meals. Of note, Saudi Arabia has not experienced any food insecurity during the pandemic, and food has been widely available in the grocery shops for everyone.

#### 4.1.6. Income

An interesting finding from our study was the relationship of EE with both baseline levels of family income and changes (increases) to it during the pandemic. Women whose family income was in the lowest tier, as well as those whose income had increased, reported a lower EES score than did women who reported no change in income or had a higher baseline. By contrast, a recent study among 150 participants from varied socioeconomic classes showed that lower socioeconomic status was linked to higher distress and emotional eating [75]. A recent study [76] proposed that socioeconomic disadvantage causes psychological and emotional distress that is transferred from parents to children, thereby fostering psychological and emotional overload in the home and leading to maladaptive coping strategies, such as are seen in EE [76].

While an increase in income might produce greater emotional security in response to greater economic security and therefore less likelihood to engage in EE, this would not explain the relatively low EE scores across the lower and extending into our middle-income tier. One possibility is that, absent the direst poverty-driven malnutrition, lower income constrains food budgets sufficiently that decisions about buying and consuming food are perceived as—and may objectively be—more rational and less emotion driven.

#### 4.1.7. Potential Interventions

Reducing maladaptive responses to stress during the COVID-19 pandemic is an immediate goal that has potential for long-term as well as short-term health benefits, including a reduction in weight gain that may be driven by EE. Apart from maladaptive eating, our study showed a high stress score was positively correlated with worse sleep and less sleep. Additionally, a high stress score correlated positively with less activity, as indicated by longer sitting time, and negatively correlated with steps per day. A previous study [77] confirmed that optimizing sleep duration can reduce stress, and that physical activity can also help improve sleep. More recently, Muscogiuri et al. pointed out that quarantine-related stress leads to sleep disturbances that further worsen stress and increase food cravings [56]. The authors also suggested potential benefits of eating foods that contain or promote the synthesis of serotonin and melatonin at dinner [56].

In synergetic ways, increased physical activity addresses multiple aspects of the stress complex. Increased physical activity has been shown to lower EE [17]. Physical activity has been linked to a reduction in stress and less consumption of unhealthy food [78]. A randomized controlled trial showed increased physical activity improving both mood and sleep [79], which may be moderated by serotonin and melatonin, respectively [80,81]. The challenge in quarantine is to increase activity while social movement and interaction are restricted, and where heightened depressive and anxious responses to stress may hinder adoption of healthier habits at home.

#### 4.1.8. Limitations

This study has several limitations. First, the study focused on King Saud University students, graduates, and alumni, who are likely women with a higher education status. King Saud university is one of the largest public university in Saudi Arabia with approximately 51,000 students. Since university students are fairly representative of young people at large and Saudi Arabia holds very high literacy rate therefore it was deemed that female university student’s sample would be ideal to represent young female population of Saudi Arabia. Furthermore, the average age of sample participants is 22.0 ± 1.9 and majority belonging to families earning more than 10,000 SAR. The participants profile of this study are not different from young female in Saudi Arabia in terms of age and socio-economic status [82]. Second, as with all studies that rely on self-reported data, our results are subject to the influence of response and recall bias. However, the degree to which this type of bias may have impacted the information gathered about weight and height may not be especially problematic, as a previous study found a strong correlation between the measured and reported values for the samples studied [83]. Third, the study lacked anonymity of the responders. Finally, the cross-sectional design of the study has several inherent limitations, and a longitudinal study would doubtless provide greater insight into how a prolonged period of quarantine impacts emotional eating.

The study has three main strengths. One is that it is the first study to assess EE during the COVID-19 pandemic. A second strength is that it focused on young women who are more likely to engage in EE and are at an increased risk of experiencing mental health disorders. The third strength is that the participants were classified using the SPSS binning option to classify low, moderate, and high emotional eaters. This contrasts with previous studies that used either the mean or the median. However, the selection of participants with extreme EE scores has been proposed as a better strategy for identifying EE behavior. Finally, the study assessed numerous covariates with adjustments made to identify independent associations.

## 5. Conclusions

Though mandatory quarantine helped control the rate of COVID-19 infection in Saudi Arabia, it was associated with an increase in EE and mental health disorders. More than half of the women sampled were emotional eaters. Our chosen methods identified a psychological determinant as the main driver of EE during the quarantine: adopting a strategy for coping with negative moods by eating more unhealthy meals and choosing fatty, energy-dense meals. That obese women tended more to be emotional eaters points to a biological determinant. Finally, chronic stress due to the pandemic represents a situational determinant that aggravated EE. Because EE increases the risk of obesity among young women and has adverse future health effects, women can lower this risk lowering their level of stress during the COVID-19 pandemic by increasing physical activity and taking dietary and behavioral steps to improve sleep quality. Furthermore, public health policies should promote awareness of healthy eating and lifestyle habits and provide additional support for women with obesity and eating disorders and associated mental health disorders. Our study identified some associations between EE and income levels and changes. Future research in this area may illuminate further opportunities for intervention.

## Figures and Tables

**Table 1:** General characteristics of study sample.

		Overall *n* (%) Mean ± SD
***n* (%)**		**638**
**Age** (in years)		22.0 ± 1.9
**BMI** (kg/m^2^)		23.2 ± 5.0
**BMI categories**		
Obese		58 (9.1%)
Overweight		115 (18.1%)
Normal		385 (60.4%)
Underweight		79 (12.4%)
**Sociodemographic**		
Education level	Bachelor	413 (64.7%)
	Internship	62 (9.7%)
	Graduate or higher	163 (25.5%)
GPA		4.3 ± 0.9
Occupation	Unemployed	579 (90.8%)
	Health Sector	8 (1.3%)
	Government non-health sector	11 (1.7%)
	Private sector	35 (5.5%)
	Business	5 (0.8%)
Family monthly income	Less than 5000 Saudi Riyals	42 (6.6%)
	5000–10,000 Saudi Riyals	116 (18.2%)
	10,000–20,000 Saudi Riyals	226 (35.4%)
	>20,000 Saudi Riyals	254 (39.8%)
Area of residency	North	259 (40.7%)
	South	72 (11.3%)
	East	139 (21.8%)
	West	115 (18.1%)
	Middle	25 (3.9%)
	Other	27 (4.2%)

Data presented as mean ± SD for continuous and *n* (%) for categorical variables; grade point average (GPA). *p*-value < 0.05 considered significant.

**Table 2:** Mental status and parameters related to COVID-19.

		Overall *n* (%) Mean ± SD
***n***		**638**
**Stress**		
Total PSS score		19.2 ± 6.2
PSS score groups	Low stress	103 (16.1)
	Moderate stress	455 (71.3)
	Severe stress	80 (12.5)
**Depression**		
Total PHQ-9 score		9.3 ± 5.4
PHQ-9 groups	No depression (<10)	365 (57.2)
	Depression (≥10)	273 (42.8)
**Anxiety**		
Total GAD-10 score		7.2 ± 4.7
GAD-10 groups	No anxiety (<10)	465 (73.0)
	Anxiety (≥10)	172 (27.0)
**Covid-19 -related parameters**		
Change in Family income	No	502 (84.4)
	Yes, Decreased	56 (9.4)
	Yes, Increased	37 (6.2)
Changing Residency	No	596 (93.4)
	Yes	42 (6.6)
Infected by COVID-19	No	637 (99.8)
	Yes	1 (0.2)
Family member infected by COVID-19	No	626 (98.1)
	Yes	12 (1.9)
Specify member	Father or Mother	3 (30.0)
	Extended family	7 (70.0)
Type of quarantine	Medical Isolation	2 (0.3)
	Self-Quarantine	636 (99.7)
Food cleaning	No	2 (0.9)
	Yes	233 (99.1)
Smoking	No	327 (95.9)
	Yes	14 (4.1)

Data presented as the mean ± SD for continuous and *n* (%) for categorical variables. PSS; Perceived Stress scale, GAD-7; Generalized Anxiety Disorder-7, PHQ-9; Patient Health Questionnaire-9. *p*-value < 0.05 considered significant.

**Table 3:** Correlations between variables.

	Total EES Score	EE-Depression	EE-Anxiety	EE-Anger	Total GAD-7 Score	Total PHQ-9 Score	Total PSS Score
Age	0.004	−0.020	0.011	0.013	0.009	−0.027	−0.023
GPA	0.097 *	0.105 **	0.075	0.069	−0.066	−0.086 *	−0.039
BMI	0.111 **	0.143 **	0.027	0.082 *	0.017	0.034	0.034
**Mental Status**							
Total PHQ-9 score	0.060	0.071	0.090 *	0.068	0.636 **	1	0.521 **
Total GAD-7 score	0.062	0.060	0.117 **	0.076	1	0.636 **	0.580 **
Total PSS score	0.130 **	0.128 **	0.129 **	0.138 **	0.580 **	0.521 **	1
**Dietary parameters**							
Number of main meals/day	0.173 **	0.168 **	0.144 **	0.116 **	−0.139 **	−0.185 **	−0.095 *
Number of snacks/day	0.080	0.077	0.079	0.062	0.025	0.028	0.084 *
Fat intake (g/day)	0.178 **	0.123	0.214 **	0.193 **	0.143 *	0.004	−0.095
Protein intake (g/day)	0.151 *	0.153 *	0.155 *	0.137 *	0.101	0.043	−0.114
CHO intake (g/day)	0.054	0.037	0.072	0.008	0.050	0.069	0.022
Energy intake (kcal/day)	0.130 *	0.103	0.161 *	0.108	0.110	0.061	−0.041
**Physical activity parameters**							
GPAQ score	−0.059	−0.055	−0.058	−0.053	0.059	−0.016	−0.003
Steps/day	−0.097	−0.067	−0.102	−0.038	−0.092	−0.149 **	−0.224 **
Sitting (min/day)	−0.002	−0.010	0.043	−0.004	0.107 **	0.086 *	0.080 *
**Sleep parameters**							
Global PSQI score	−0.021	−0.010	0.027	−0.004	0.178 **	0.245 **	0.159 **
Duration of sleep (h/day)	0.016	0.012	0.016	0.000	−0.210 **	−0.180 **	−0.196 **

Data presented as Pearson correlation coefficient, with significant correlations in bold; ** and * indicate significance at 0.01 and 0.05 respectively. Body mass index, BMI; Grade point average, GPA; Perceived Stress Questionnaire, PSS; Generalized Anxiety Disorder-7, GAD-7; Patient Health Questionnaire-9, PHQ-9; and Pittsburgh Sleep Quality Index, PSQI.

**Table 4:** Indicators and Predictors of EES score among young women during mandatory quarantine.

Parameters	Unadjusted			Adjusted			Adj. R^2^	F(10,566)
	B ± SE	Std. Beta	*p*-Value	B ± SE	Std. Beta	*p*-Value		
**Age**	0.0 ± 0.3	0.004	0.923	0.1 ± 0.3	0.006	0.874	--	
**BMI**	0.5 ± 0.1	0.153	0.000	0.5 ± 0.1	0.149	<0.001	--	
**Income** (<5000 Saudi Riyals)	−0.6 ± 2.6	−0.009	0.815	−1.3 ± 2.6	−0.019	0.625	--	
**Graduate or higher**	−1.1 ± 1.5	−0.029	0.471	−1.0 ± 1.5	−0.028	0.481	0.05	3.9
**Unemployed**	0.9 ± 2.2	0.015	0.697	1.1 ± 2.2	0.020	0.618	0.05	3.9
**Number of children**	1.9 ± 1.2	0.123	0.106	2.2 ± 1.2	0.139	0.065	0.08	2.4
**Change in family income**							0.05	3.3
Increased	−5.2 ± 2.6	−0.080	0.051	−5.2 ± 2.6	−0.081	0.049	--	
Decreased	0.9 ± 2.2	0.016	0.689	0.3 ± 2.2	0.006	0.881	--	
No change	Reference						--	
**COVID-19 infection in family**	0.1 ± 4.5	0.001	0.978	−0.1 ± 4.5	−0.001	0.979	0.05	3.9
**Total PHQ-9 score**	0.2 ± 0.1	0.060	0.130	0.0 ± 0.2	0.006	0.917	0.05	4.3 *
**Total GAD-7 score**	0.2 ± 0.1	0.062	0.119	0.0 ± 0.2	−0.003	0.958	0.05	4.3 *
**Total PSS score**	0.3 ± 0.1	0.130	0.001	0.4 ± 0.1	0.143	0.004	0.05	4.3 *
**Number of meals**	4.1 ± 1.0	0.173	0.000	4.5 ± 1.0	0.187	<0.001	0.05	4.3 *
**Fast food intake**							0.06	4.0
At least 2 to 4 times a week	4.6 ± 1.5	0.136	0.003	4.3 ± 1.5	0.127	0.005	--	
1 to 4 in a month	6.0 ± 2.4	0.114	0.012	5.9 ± 2.4	0.111	0.014	--	
Once or no in a month	Reference						--	
**Sugary food consumptio** **n**	2.5 ± 0.6	0.155	0.000	2.4 ± 0.6	0.150	<0.001	0.07	5.1
**Fat intake (g/day)**	0.0 ± 0.0	0.178	0.006	0.0 ± 0.0	0.192	0.004	0.07	2.7
**Carbohydrate intake (g/day)**	0.0 ± 0.0	0.054	0.408	0.0 ± 0.0	0.055	0.404	0.04	1.9
**Energy intake (kcal/day)**	0.0 ± 0.0	0.130	0.046	0.0 ± 0.0	0.134	**0.043**	0.05	2.3
**Global PSQI score**	−0.1 ± 0.2	−0.021	0.599	−0.3 ± 0.2	−0.065	0.127	0.05	4.3 *
**GPAQ SCORE**	0.0 ± 0.0	−0.059	0.135	0.0 ± 0.0	−0.061	0.124	0.05	4.3 *
**Sitting (min/day)**	0.0 ± 0.0	−0.002	0.959	0.0 ± 0.0	−0.006	0.878	0.05	3.9
**Steps/day**	0.0 ± 0.0	−0.097	0.074	0.0 ± 0.0	−0.068	0.221	0.06	3.1 **

Data presented as B ± SE from linear regression; *p*-value adjusted for age, BMI, income, diet, PHQ-9, GAD-7, PSS, PSQI, and physical activity; *p* < 0.05 was considered statistically significant. * & ** indicates F-statistics for F (9567) and F (10,329).

## Supplementary Materials

The following are available online at https://www.mdpi.com/2072-6643/12/10/2923/s1, Table S1: Characteristics of study sample according to EE group. Table S2: General mental status and parameters related to COVID-19 and according to EES groups. Table S3: Nutrition, physical activity, and sleep parameters according to EE groups.
