# Supplementary material for: Prevalence and Predictors of Emotional Eating among Healthy Young Saudi Women during the COVID-19 Pandemic
Source: Nutrients. 2020 Sep 24;12(10):2923. doi: 10.3390/nu12102923 (PMC7598723; doi:10.3390/nu12102923)
Supplement: Supplementary file 1 [file nutrients-12-02923-s001.zip › =Supplementary file 1-Tables-18 sep.docx]

**Supplementary Table 1.** Characteristics of study sample according to EE group

|  | | **Emotional Eating (EE) categories** | | |  |
| --- | --- | --- | --- | --- | --- |
|  |  | **Low EE**  **≤27.5** | **Moderate EE**  **27.6-43.6** | **High EE**  **≥43.7** | **P-values** |
| N (%) | | 335 (52.5) | 202 (31.7) | 101 (15.8) |  |
| **Age** (in years) | | 22.1 ± 1.9 | 21.7 ± 1.7 | 22.3 ± 2.3 | 0.006 |
| **BMI** (kg/m^2^) | | 22.7 ± 4.9 | 23.4 ± 6.4 | 25.4 ± 6.3 | <0.001 |
| **BMI categories** | |  |  |  |  |
| Obese | | 21 (6.3) | 20 (9.9) | 17 (16.8) | 0.003 |
| Overweight | | 56 (16.8) | 35 (17.3) | 24 (23.8) |  |
| Normal | | 208 (62.3) | 120 (59.4) | 57 (56.4) |  |
| Underweight | | 49 (14.7) | 27 (13.4) | 3 (3.0) |  |
| **Sociodemographic** |  |  |  |  |  |
| Education level | Bachelor | 209 (62.4) | 134 (66.3) | 70 (69.3) | 0.338 |
|  | Internship | 39 (11.6) | 18 (8.9) | 5 (9.7) |  |
|  | Graduate or higher | 87 (26.0) | 50 (24.8) | 26 (25.8) |  |
| GPA | | 4.2 ± 1.0 | 4.4 ± 0.8 | 4.3 ± 0.8 | 0.275 |
| Occupation | Unemployed | 305 (91.0) | 181 (89.6) | 93 (92.1) | 0.522 |
|  | Health Sector | 2 (.6) | 4 (2.0) | 2 (2.0) |  |
|  | Government non-health sector | 8 (2.4) | 2 (1.0) | 1 (1.0) |  |
|  | Private sector | 19 (5.7) | 12 (5.9) | 4 (4.0) |  |
|  | Business | 1 (.3) | 3 (1.5) | 1 (1.0) |  |
| Family monthly income |  |  |  |  | 0.090 |
|  | Less than 5000 Saudi Riyals | 22 (7.3) | 14 (5.4) | 6 (7.6) |  |
|  | 5000–10000 Saudi Riyals | 51 (16.9) | 59 (22.9) | 6 (7.6) |  |
|  | 10000–20000 Saudi Riyals | 104 (34.6) | 89 (34.5) | 33 (41.8) |  |
|  | >20000 Saudi Riyals | 124 (41.2) | 96 (37.2) | 34 (43.0) |  |
| Area of residency | North | 134 (40.1) | 81 (40.1) | 44 (43.6) | 0.691 |
|  | South | 35 (10.5) | 25 (12.4) | 12 (11.9) |  |
|  | East | 74 (22.2) | 41 (20.3) | 24 (23.8) |  |
|  | West | 63 (18.9) | 37 (18.3) | 15 (14.9) |  |
|  | Middle | 16 (4.8) | 5 (2.5) | 4 (4.0) |  |
|  | Other | 12 (3.6) | 13 (6.4) | 2 (2.0) |  |
|  |  |  |  |  |  |

Data presented as mean ± SD for continuous and N (%) for categorical variables; grade point average (GPA). P-value < 0.05 considered significant.

**Supplementary Table 2.** General mental status and parameters related to COVID-19 and according to EES groups.

|  | | | **Emotional Eating (EE) categories** | | | |  |
| --- | --- | --- | --- | --- | --- | --- | --- |
|  |  |  | **Low EE**  **≤27.5** | **Moderate EE**  **27.6-43.6** | **High EE**  **≥43.7** | **P-values** | |
| N | | | 335 (52.5) | 202 (31.7) | 101 (15.8) |  | |
| **Stress** | | |  |  |  |  | |
| Total PSS score | | | 18.7 ± 6.5 | 19.4 ± 5.8 | 20.3 ± 5.9 | 0.066 | |
| PSS score groups | Low stress | | 64 (19.1) | 27 (13.4) | 12 (11.9) | 0.130 | |
|  | Moderate stress | | 233 (69.6) | 151 (74.8) | 71 (70.3) |  |  |
|  | Severe stress | | 38 (11.3) | 24 (11.9) | 18 (17.8) |  |  |
| **Depression** | | |  |  |  |  | |
| Total PHQ-9 score | | | 9.3 ± 5.7 | 9.0 ± 5.0 | 10.0 ± 5.3 | | 0.266 |
| PHQ-9 groups | | No depression (<10) | 187 (55.8) | 126 (62.4) | 52 (51.5) | | 0.148 |
|  |  | Depression (≥10) | 148 (44.2) | 76 (37.6) | 49 (48.5) | |  |
| **Anxiety** | | |  |  |  | |  |
| Total GAD-10 score | | | 7.0 ± 5.0 | 7.3 ± 4.4 | 7.5 ± 4.5 | | 0.526 |
| GAD-10 groups | | No anxiety (<10) | 244 (73.1) | 147 (72.8) | 74 (73.3) | | 0.995 |
|  |  | Anxiety (≥10) | 90 (26.9) | 55 (27.2) | 27 (26.7) | |  |
| **Covid-19 -related parameters** | | |  |  |  | |  |
| Change in Family income | | No | 257 (82.1) | 168 (88.0) | 77 (84.6) | | 0.068 |
|  |  | Yes, Decreased | 29 (9.3) | 19 (9.9) | 8 (8.8) | |  |
|  |  | Yes, Increased | 27 (8.6) | 4 (2.1) | 6 (6.6) | |  |
| Changing Residency | | No | 313 (93.4) | 187 (92.6) | 96 (95.0) | | 0.715 |
|  |  | Yes | 22 (6.6) | 15 (7.4) | 5 (5.0) | |  |
| Infected by COVID-19 | | No | 335 (100.0) | 201 (99.5) | 101 (100.0) | | 0.339 |
|  |  | Yes | 0 (0.0) | 1 (.5) | 0 (0.0) | |  |
| Family member infected by COVID-19 | | No | 328 (97.9) | 198 (98.0) | 100 (99.0) | | 0.769 |
|  |  | Yes | 7 (2.1) | 4 (2.0) | 1 (1.0) | |  |
| Specify member | | Father or Mother | 3 (50.0) | 0 (0.0) | 0 (0.0) | | 0.240 |
|  |  | Extended family | 3 (50.0) | 3 (100.0) | 1 (100.0) | |  |
| Type of quarantine | | Medical Isolation | 2 (0.6) | 0 (0.0) | 0 (0.0) | | 0.404 |
|  |  | Self-Quarantine | 333 (99.4) | 202 (100.0) | 101 (100.0) | |  |
| Food cleaning | | No | 0 (0.0) | 1 (1.0) | 1 (2.9) | | 0.281 |
|  |  | Yes | 99 (100.0) | 100 (99.0) | 34 (97.1) | |  |
| Smoking | | No | 197 (97.0) | 80 (95.2) | 50 (92.6) | | 0.322 |
|  |  | Yes | 6 (3.0) | 4 (4.8) | 4 (7.4) | |  |

Data presented as the mean ± SD for continuous and N (%) for categorical variables. PSS; Perceived Stress scale, GAD-7; Generalized Anxiety Disorder-7, PHQ-9; Patient Health Questionnaire-9. P-value < 0.05 considered significant.

**Supplementary Table 3.** Nutrition, physical activity, and sleep parameters according to EE groups.

|  | | **Emotional Eating (EE) categories** | | | |  |
| --- | --- | --- | --- | --- | --- | --- |
|  |  | **Low EE**  **≤27.5** | **Moderate EE**  **27.6-43.6** | | **High EE**  **≥43.7** | **P-values** |
| **Nutrition related parameters** | |  | |  |  |  |
| Weight changes | Increased | 82 (24.5) | | 62 (31.0) | 34 (33.7) | 0.281 |
|  | Decreased | 115 (34.3) | | 58 (29.0) | 31 (30.7) |  |
|  | No change | 138 (41.2) | | 80 (40.0) | 36 (35.6) |  |
| Following a weight loss diet | No | 277 (83.2) | | 175 (87.1) | 76 (76.0) | 0.053 |
|  | Yes | 56 (16.8) | | 26 (12.9) | 24 (24.0) |  |
| Number of main meals/day | | 1.9 ± 0.7 | | 2.0 ± 0.7 | 2.1 ± 0.6 | 0.001 |
| Number of snacks/day | | 1.8 ± 1.0 | | 1.9 ± 1.0 | 2.0 ± 1.1 | 0.138 |
| Sugary food consumption | Usually | 77 (23.0) | | 51 (25.2) | 40 (39.6) | 0.001 |
|  | Often | 63 (18.8) | | 41 (20.3) | 15 (14.9) |  |
|  | Sometimes | 160 (47.8) | | 98 (48.5) | 42 (41.6) |  |
|  | Rarely | 26 (7.8) | | 10 (5.0) | 4 (4.0) |  |
|  | Never | 9 (2.7) | | 2 (1.0) | 0 (0.0) |  |
| Sugar Craving | Usually | 79 (23.6) | | 53 (26.2) | 36 (35.6) | 0.005 |
|  | Often | 55 (16.4) | | 37 (18.3) | 18 (17.8) |  |
|  | Sometimes | 169 (50.4) | | 101 (50.0) | 42 (41.6) |  |
|  | Rarely | 24 (7.2) | | 8 (4.0) | 4 (4.0) |  |
|  | Never | 8 (2.4) | | 3 (1.5) | 1 (1.0) |  |
| Fast food intake | No | 200 (66.4) | | 104 (55.9) | 46 (50.5) | 0.007 |
|  | Yes | 101 (33.6) | | 82 (44.1) | 45 (49.5) |  |
| Fast food frequency intake | Less than one per month or zero | 169 (60.6) | | 72 (45.9) | 39 (45.9) | 0.017 |
|  | 1–4 times a month | 84 (30.1) | | 68 (43.4) | 34 (40.0) |  |
|  | At least 2–4 times a week | 26 (9.3) | | 17 (10.7) | 12 (14.1) |  |
|  |  |  | |  |  |  |
| Water intake (mL/day) | <500 | 49 (24.1) | | 11 (13.1) | 5 (9.3) | 0.005 |
|  | 1000–1500 | 101 (49.8) | | 46 (54.8) | 23 (42.6) |  |
|  | >1600 | 53 (26.1) | | 26 (31.0) | 26 (48.2) |  |
| Fat intake (g/day) | | 109.6 ± 60.0 | | 130.5 ± 91.5 | 141.7 ± 122.2 | 0.089 |
| Protein intake (g/day) | | 88.5 ± 42.0 | | 98.6 ± 59.3 | 99.7 ± 52.9 | 0.315 |
| Carbohydrate intake (g/day) | | 301.0 ± 143.4 | | 345.7 ± 366.7 | 318.9 ± 153.3 | 0.489 |
| Energy (kcal/day) | | 2372.3 ± 1073.8 | | 2671.2 ± 1790.6 | 2744.4 ± 1592.6 | 0.268 |
| **Physical activity parameters** | |  | |  |  |  |
| Sitting (min/day) | | 539.4 ± 737.9 | | 515.1 ± 278.4 | 505.4 ± 303.9 | 0.823 |
| Total GPAQ score | | 1988.2 ± 13463.8 | | 885.9 ± 1326.3 | 1598.1 ± 2065.8 | 0.453 |
| Daily steps | | 2987.6 ± 4559.3 | | 2434.0 ± 2484.5 | 2555.7 ± 3486.6 | 0.508 |
| **Sleep parameters** | |  | |  |  |  |
| Global PSQI score | | 7.5 ± 8.6 | | 6.8 ± 3.9 | 6.7 ± 3.8 | 0.393 |

Data presented as mean ± SD for continuous and N (%) for categorical variables; P-value < 0.05 considered significant. GPAQ; Global Physical Activity Questionnaire and PSQI; Pittsburgh Sleep Index.
