# Supplementary material for: Prevalence and Predictors of Emotional Eating among Healthy Young Saudi Women during the COVID-19 Pandemic
Source: Nutrients. 2020 Sep 24;12(10):2923. doi: 10.3390/nu12102923 (PMC7598723; doi:10.3390/nu12102923)
Supplement: Supplementary file 1 [file nutrients-12-02923-s001.zip › =Supplementary file 2-Questionnaire.pdf]

Evaluating the healthy lifestyle for King Saud University during Covid-19 pandemic in Kingdom of Saudi Arabia

Dear participants,
Thank you for agreeing to participate in this electronic survey, which consider a part of a research done by a research team from King Saud University.

The purpose of this survey is to evaluate your healthy lifestyle, like your physical activity, and your sleep during the current conditions due to Covid-19 pandemic. As well as, to evaluate your psychological status during this conditions by using a depression and anxiety indexes, and to evaluate your emotional eating status. Participation is available for King Saud University students.

As a participant, you will get:
- Degree of anxiety and depression
- Emotional eating status

Please, write your email so the research team can inform you about your degree.
Your answers will help you a lot in controlling quantity and quality of your food.

All questions will depend on the period of quarantine.

Assure that your answers will be kept strictly confidential and only used for research purpose.
If you agree in participating with this survey, please click “next” to start.

تقييم أسلوب صحة الحياة لدى طلاب جامعة الملك سعود أثناء جائحة كورونا المستجد في المملكة العربية السعودية

عزيزي المشارك /عزيزتي المشاركة
شكرا على موافقتكم على المشاركة في هذا الاستبيان الإلكتروني الذي هو جزء من أبحاث يقوم بها فريق بحثي من جامعة الملك سعود.
الهدف هو تقييم أسلوب صحة حياتك مثل نشاطك و نومك في هذه الظروف الراهنة بسبب وباء كورونا. كذلك تقييم وضعك النفسي في هذه الظروف من خلال مؤشرالاكتئاب والقلق العام بالإضافة لتقييم مستوى الشراهة في الأكل. المشاركة متاحة لطلاب و طالبات جامعة الملك سعود
.

كمشترك/ة في البحث، ستحصل على نتائج:
-درجة القلق و الاكتئاب.
- ما إذا كان عواطفك تؤثر على طعامك؟ "لا تجعل نفسيتك تتحكم بكمية و نوع طعامك".

أرجو وضع ايميلك ليقوم الفريق البحثي بإبلاغك بنتيجة الإجابة باذن الله.
هذه الأجوبة ستساعدك كثيرا في التحكم بكمية و نوع طعامك.

دقة و مصداقية إجابتك عنصر مهم لنجاح البحث. سيستغرق تعبئتة هذا الاستبيان ٢٠ دقيقه من وقتك.
ومشاركتك معنا تعتبر صدقة جارية عن علمك إن شاء الله و خدمة لوطنك.

*الأسئلة تعتمد جميعها خلال فترة العزل.

نؤكد لك أن جميع إجاباتك ستبقى في سرية تامة و لأغراض البحثية فقط.
إذا كنت موافق/ة على المشاركة في هذا الاستبيان الإلكتروني، أرجو النقر على "التالي" للبدء.

* Required

1. هل أنت طالب/ة أو متخرج/ة جامعة الملك سعود ؟ * Are you student/graduate from King Saud university?

Mark only one oval.

- نعم Yes
- لا No

2. هل أنت سعودي/ة الجنسية ؟ * Are you Saudi ?

Mark only one oval.

- نعم Yes
- لا No

Untitled Section

3. هل عمرك ضمن 18-39 سنة ؟ *

Is your age within 18-39 years old?

Mark only one oval.

- ⬭ نعم — Yes
- ⬭ لا — No

4. هل تم تشخيصك من قِبل طبيب بأي من الآتي؟ *

Have you ever been diagnosed by a physician with:

Mark only one oval.

- ⬭ لا أعاني من أي منها — None
- ⬭ مرض السكري و سكر الحمل — DM, GDM
- ⬭ مشاكل في الغدة الدرقية (نشاط او خمول) — Hypo/hyperthyroidism
- ⬭ حامل/مرضعة — Pregnant/lactating women
- ⬭ أمراض الجهاز الهضمي (مثل القولون و غيره) — Digestive system diseases
- ⬭ مشاكل بالنوم او مشاكل نفسية مشخص من قبل طبيب و/أو تم أخذ الدواء للحالة — Psychological or sleep disorders
- ⬭ وجود بروتين في البول — Proteinuria
- ⬭ الداء النشواني — Amyloidosis
- ⬭ التهاب المفاصل — Arthritis
- ⬭ تضخم اللوزتين أو اللحمية — Enlarged tonsils or adenoids
- ⬭ سوء امتصاص — Malabsorption
- ⬭ داء الانسداد الرئوي المزمن — COPD
- ⬭ أورام — Tumors
- ⬭ أمراض القلب المزمن (مثل انسداد الشرايين و غيره) — Heart diseases
- ⬭ أمراض الكلى المزمن (مثل غسيل الكلوي، الحصوات و غيره) — Kidney diseases
- ⬭ أنيميا (فقر الدم) مشخص من قبل طبيب و/أو تم أخذ الدواء للحالة — Anemia
- ⬭ اضطراب عمليات الأيض — Metabolic syndrome
- ⬭ ارتفاع الدهون الثلاثية — Hyper triglycerides
- ⬭ ارتفاع ضغط الدم — Hypertension
- ⬭ ضعف تحمل الجلوكوز — Glucose intolerance
- ⬭ هشاشة العظام — Osteoporosis
- ⬭ الربو — Asthma

البيانات الشخصية

5. * الاسم Name

6. الايميل Email

7. * رقم الجوال Mobile number

8. * العمر Age

9. * مكان الميلاد Birth place

10. * تاريخ الميلاد (بالميلادي) ؛ السنة/اليوم/الشهر Birth date

Example: January 7, 2019

11. * الجنس Gender

Mark only one oval.

- ( ) ذكر Male
- ( ) أنثى female

التاريخ المرضي الشخصي Personal medical history

12. * عمر البلوغ Puberty age

13. إذا كنت أنثى، هل الدورة الشهرية

If female, is your menstrual cycle

Mark only one oval.

- منتظمة — Regular
- غير منتظمة — Irregular

التاريخ المرضي العائلي

Family medical history

14. هل يوجد هذه الأمراض في الوالد أو الوالدة أو الجد أو الجدة ؟ *

Do your parents or grandparents have one of these diseases

Mark only one oval per row.

	نعم Yes.	لا No
ارتفاع ضغط الدم — Hypertension		
ارتفاع الدهون الثلاثية — Hyper triglycerides		
أمراض القلب — Heart diseases		
هشاشة العظام — Osteoporosis		
الربو — Asthma		
السرطان — Cancer		
أمراض الكبد — Liver diseases		
أمراض الكلى — Kidney diseases		

15. في حال وجود أمراض غير ذلك الرجاء ذكرها

In case there were other diseases, please mention them

____

16. السكري ؛ بإمكانك الخيار أكثر من شخص * DM

Check all that apply.

- [ ] لا يوجد
- [ ] الأم
- [ ] الأب
- [ ] الأخ/الأخت
- [ ] الأولاد
- [ ] جد/جدة
- [ ] عم/عمة
- [ ] خال/خالة

None
Mother
Father
Brother/sister
Children
Grandparents
Uncle
Aunt

17. سكر الحمل ؛ بإمكانك الخيار أكثر من شخص * GDM

Check all that apply.

- [ ] لا يوجد
- [ ] الأم
- [ ] الأخت
- [ ] الابنة
- [ ] جدة
- [ ] عمة
- [ ] خالة

None
Mother
Father
Brother/sister
Children
Grandparents
Uncle
Aunt

18. السمنة ؛ بإمكانك الخيار أكثر من شخص * Obesity

Check all that apply.

- [ ] لا يوجد
- [ ] الأم
- [ ] الأب
- [ ] الأخ/الأخت
- [ ] الأولاد
- [ ] جد/جدة
- [ ] عم/عمة
- [ ] خال/خالة

None
Mother
Father
Brother/sister
Children
Grandparents
Uncle
Aunt

البيانات الاجتماعية الديموغرافية Socio-demographic information

19. 1-القسم * Department

Mark only one oval.

- صحي — Health
- علمي — Science
- إنساني — Humanity

20. 2-الكلية؛ إذا كانت الإجابة أخرى الرجاء كتابة الكلية في خانة (other) * College

Mark only one oval.

- كلية الآداب — College of arts
- كلية التربية — College of education
- كلية إدارة الأعمال — College of Business Administration
- كلية اللغات و الترجمة — College of Languages and Translation
- كلية العلوم — College of Science
- كلية علوم الحاسب و المعلومات — College of Computer and Information Sciences
- كلية الحقوق و العلوم السياسية و كلية السياحة و الآثار — College of Law, Political Sciences, Tourism and Archeology
- كلية الصيدلة — College of Pharmacy
- كلية طب الأسنان — College of Dentistry
- كلية العلوم الطبية التطبيقية — College of Applied Medical Sciences
- كلية الطب — College of Medicine
- كلية التمريض — College of Nursing
- كلية علوم الرياضة و النشاط البدني — College of Sport Sciences and Physical Activity
- كلية علوم الأغذية و الزراعة — College of Food and Agricultural Sciences
- كلية الهندسة — College of Engineering
- كلية العمارة و التخطيط — College of Architecture and Planning
- كلية المجتمع — Community College
- كلية الدراسات التطبيقية و خدمة المجتمع — College of Applied Studies and Community Services
- كلية الأمير سلطان بن عبدالعزيز للخدمات الطبية الطارئة — Prince Sultan Bin Abdulaziz College for Emergency Medical Services
- Other: ______

21. 3-المستوى التعليمي * Educational level

Mark only one oval.

- طالب/ة بكالوريس — Bachelor degree
- تدريب ميداني/ امتياز — Internship
- طالب/ة ماجستير — Master level
- طالب/ة دكتوراه — PHD
- متخرج/ة — Graduated

If you were a student, specify your level

22. 4-إذا كنت طالب/ة أي مستوى ؟ و إذا كانت الإجابة (طالب/ة امتياز أو متخرج/ة) اختار (لا) *

Mark only one oval per row.

	لا	1	2	3	4	5	6	7	8	9
المستوى الدراسي										

23. 5-المعدل التراكمي * GPA

Mark only one oval.

- وأقل 3 — Less than 3
- أكثر من 3 إلى 3.5 — More than 3 to 3.5
- أكثر من 3.5 إلى 4 — More than 3.5 to 4
- أكثر من 4 إلى 4.5 — More than 4 to 4.5
- أكثر من 4.5 إلى 5 — More than 4.5 to 5

24. 6-الوظيفة * Job

Mark only one oval.

- غير موظف/ة — Unemployed
- موظف/ة في قطاع صحي — Health sector
- (موظف/ة في قطاع حكومي) في جهة غير صحية — Governmental sector
- موظف/ة في قطاع خاص — Private sector
- رجل/سيدة أعمال) أعمال حرة) — Business man/woman

25. 7- دخل الأسرة الشهري * Family income

Mark only one oval.

- ليس لدينا دخل — No income
- أقل من 5000 ريال — Less than 5000 RS
- 5000-10000 ريال — 5000-10000 RS
- 10000-20000 ريال — 10000-20000 RS
- أكثر من 20000 ريال — More than 20000 RS

26. 8-هل تغير دخل الأسرة خلال فترة العزل؟ * Did the family income changed during quarantine

Mark only one oval.

- لم يتغير — No change
- نعم، زاد الدخل — Yes increased
- نعم، نقص الدخل — Yes decreased

27. 9-الحالة الاجتماعية * Marital status

Mark only one oval.

- متزوج/ة — Married
- مطلق/ة — Divorced
- أرمل/ة — Widower
- أعزب/عزباء — Single

28. 9A- هل لديك أطفال ؟ * Do you have children

Mark only one oval.

- لا — No
- نعم — Yes

29. 9B-(كم عدد أطفالك ؟ إذا كانت الإجابة (لا) اكتب 0) * If yes, how many?

____

30. 10-منطقة السكن؛ إذا كانت الإجابة أخرى الرجاء كتابة المنطقة في خانة (other) *

Mark only one oval.

Place of residence

- شمال الرياض — North of riyadh
- جنوب الرياض — South of riyadh
- شرق الرياض — East of riyadh
- غرب الرياض — West of riyadh
- وسط الرياض — Other
- Other: ______

31. 11-نوع السكن؛ إذا كانت الإجابة أخرى الرجاء كتابة السكن في خانة (other) *

Mark only one oval.

Type of resistance

- شقة — Department
- فيلا — Villa
- دور — Floor
- سكن الطلاب — Student housing
- سكن أعضاء هيئة التدريس — Faculty housing
- Other: ______ Other

32. 12-هل يوجد مساحة خارجية في المنزل ؟ * Do you have outdoor

Mark only one oval.

- نعم — Yes
- لا — No

33. 13-مجموع عدد أفراد الأسرة الذين يسكنون معك و أنت من ضمنهم مع العاملة المنزلية *

Number of family members who live with you including housekeeper if present

______

34. 13A- عدد الكبار و المراهقين 10 سنوات و أعلى و أنت من ضمنهم ما عدا العاملة المنزلية *

Of whom adults or teenagers ?

______

35. 13B- 0) اكتب (لا يوجد) إذا كانت الإجابة ؛ عدد الرضع و الأطفال 9 سنوات و أقل) *

Of whom children of less than 9 years old

36. 13C- 0) اكتب (لا يوجد) إذا كانت الإجابة ؛ عدد عاملات المنزل) *

Of whom housekeepers

37. 15-مكان الإقامة الحالية * Current place of resistance

Mark only one oval.

- ( ) المدينة City
- ( ) القرى Village

بيانات عن الصحة العامة و الغذاء Information about general health and nutrition

38. 1-هل غيرت منطقة السكن منذ بدء الحجر الصحي؟ * Did you change the place of residence since quarantine started

Mark only one oval.

- ( ) نعم Yes
- ( ) لا No

39. 2-هل أصبت بالكورونا ؟ * have you got infected by corona virus

Mark only one oval.

- ( ) نعم Yes
- ( ) لا No

40. 3-هل أصاب أحد من القرابة الدرجة الأولى ؟ * Has some of your first degree relatives got infected by corona virus

Mark only one oval.

- ( ) نعم Yes
- ( ) لا No

41. من الذي أصاب من القرابة الدرجة الأولى؟ بإمكانك الخيار أكثر من شخص *

Check all that apply.

If yes, specify

- [ ] لا يوجد — None
- [ ] جد — Grandfather
- [ ] جدة — Grandmother
- [ ] أب — Father
- [ ] أم — Mother
- [ ] أخت — Sister
- [ ] أخ — Brother
- [ ] عم — Uncle
- [ ] عمة — Aunt
- [ ] خال
- [ ] خالة

42. 4-نوعية العزل *

Mark only one oval.

Quarantine type

- ( ) عزل صحي (في أحد ضيافات وزارة الصحة أو مستشفى) — Health isolation
- ( ) عزل منزلي — Self-quarantine

43. 5-منذ متى مدة العزل *

Mark only one oval.

Quarantine period

- ( ) أول خبر عن كورونا — Since covid-19 started
- ( ) العزل الجزئي — Since partial quarantine started
- ( ) العزل الكامل — Since full quarantine started

44. 6a- الوزن الحالي (كجم) *

Current weight in Kg

____

45. 6b-كم طولك؟ (سم) *

Height in cm

____

46. * كيف تصف وزنك الحالي مقارنة بما قبل العزل ؟-7

How you describe your current weight in compare with to your weight before quarantine

Mark only one oval.

- زاد الوزن — Increase
- نقص الوزن — Decrease
- لم يتغير الوزن — No change

47. * هل تتبع أي حمية في الوقت الحالي (خلال فترة العزل) ؟-8

Do you follow any diet currently (during quarantine period)

Mark only one oval.

- نعم — Yes
- لا — No

48. ما نوع الحمية؟ إذا كانت الإجابة(لا) اكتب (لا يوجد)

If yes, specify what hind of diet

____

49. * (عدد الوجبات يوميا (خلال فترة العزل-9

Number of daily meals during quarantine period

Mark only one oval per row.

		0	1	2	3	4
Main meals	االوجبات الرئيسية					
Snacks	الوجبات الخفيفة					

50. خلال الشهور الثلاث الماضية (خلال فترة العزل) هل أخذت مكملات غذائية مثل الفيتامينات أو المعادن؟-10 *

Check all that apply. During the last 3 months (during quarantine period), did you take any supplements

- [ ] لا
- [ ] Formula/protein supplements or bars مكمل غذائي/بروتينات
- [ ] فيتامينات منوعة Multivitamin
- [ ] فيتامين د Vitamin D
- [ ] فيتامين سي Vitamin C
- [ ] فيتامين ب١٢ Vitamin B12
- [ ] حمض الفوليك Folic acid
- [ ] الحديد Iron
- [ ] زيت كبد الحوت Cod liver oil
- [ ] أخرى؛ الرجاء ذكرها في خانة others

Other: [ ] ____________________

51. إذا كانت الإجابة بنعم، ما هو نوع المكمل الغذائي (بما في ذلك من الاسم/العلامة التجارية) و كم الكمية المستهلكة في الغالب؟

If yes, what kind of supplement (name/ trademark), and specify the consumed quantity

____

____

____

____

____

52. تناول الفيتامينات أو المعادن * Consuming supplement

Mark only one oval.

- ( ) لا أتناول No
- ( ) منتظم/ة Regularly
- ( ) غير منتظم/ة Irregularly

53. 11-مكان تناول الطعام؛ بإمكانك الخيار أكثر من مكان * Place of eating

Check all that apply.

- [ ] طاولة الطعام — Dining table
- [ ] المطبخ — Kitchen
- [ ] غرفة المعيشة — Living room
- [ ] غرفة النوم — Bedroom
- [ ] أخرى؛ الرجاء كتابة المكان في خانة أخرى (other) — Other, specify

Other: [ ] ____________________

54. 12-a-هل تطبخ؟ * Do you cook

Mark only one oval.

- ( ) نعم — Yes
- ( ) لا — No

55. 12-b-من يقوم بمهام الطبخ؟ بإمكانك الخيار أكثر من شخص * Who is in charge of cooking

Check all that apply.

- [ ] أنت — You
- [ ] الأم — Mother
- [ ] الأخت — Sister
- [ ] العاملة المنزلية — Housekeepers
- [ ] أخرى؛ الرجاء كتابة الشخص في خانة أخرى (other) — Other, specify

Other: [ ] ____________________

56. 13-هل تطلب من المطاعم (خلال فترة العزل) ؟ * Do you order from restaurants during quarantine period

Mark only one oval.

- ( ) نعم — Yes
- ( ) لا — No

57. إن كانت طلباتك من المطاعم قد قلت عن قبل العزل، فما هو السبب؟ بإمكانك الخيار أكثر من مرة-14 *

Check all that apply.

If your eating from restaurants have been decreased in comparison to before quarantine, what would be the reason

- [ ] الخوف من عدوى الكورونا
- [ ] ضيق الوقت بسبب حظر التجول
- [ ] لم تقل الطلبات عن السابق

Fear from coronal virus
Time limits due to quarantine
No change

58. ما هو معدل استهلاكك للوجبات خارج المنزل سواء من مطاعم الوجبات السريعة أو غيرها (خلال فترة العزل) ؟-15 *

Mark only one oval per row.

How often do you eat from restaurants during quarantine

	مرة باليوم	2-3 باليوم	مرة بالأسبوع	2-4 بالأسبوع	5-6 بالأسبوع	1-3 بالشهر	أقل من مرة بالشهر/ بتاتا
وجبات خارج المنزل	○	○	○	○	○	○	○

Once a day 2-3 time a day Once a week 2-4 a week 5-6 a week 1-3 per month les than once per month/never

59. هل أنت نباتي/ة ؟-16 *

Mark only one oval.

Are you vegan

- ○ لا No
- ○ Vegan لا يوجد منتجات اللحوم و الألبان و البيض
- ○ Lacto vegetarian لا يوجد منتجات اللحوم و البيض
- ○ Ovo vegetarian لا يوجد منتجات اللحوم و الألبان
- ○ Lacto ovo vegetarian لا يوجود منتجات اللحوم فقط
- ○ Pescatarian لا يوجد منتجات اللحوم و لكن يتناولون المأكولات البحرية
- ○ Other: ____________

60. 17-مستوى الشراهة للأغذية و المشروبات * Degree of craving to food and beverages

الأسئلة تعتمد جميعها خلال فترة العزل.

Mark only one oval per row.

	Very often أكثر بكثير	Often أقل بكثير	Sometimes أحيانا	Almost never شبه بتاتا	Never بتاتا
مستوى الشهية للأغذية السكرية مثل (المثلجات، الكوكيز، الشكولاته) بالمقارنه بما قبل العزل؟ Carving to Sugary foods					
أتناول الأغذية السكرية Eating sugary foods					
أشرب القهوة Drinking coffee					
أشرب الشاي Drinking tea					
أتناول مشروبات تقوية المناعة و تعزيز الصحة (الكركم-زنجبيل-الليمون) Immune boosting beverages					
أتناول العسل Consuming honey					

61. 21- معدل استهلاك المشروبات *

الأسئلة تعتمد جميعها خلال فترة العزل.

Mark only one oval per row.

		Once a day مرة باليوم	2-3 a day 2-3 باليوم	4-5 a day 4-5 باليوم	More than 6 a day أكثر من 6 باليوم	Once a week مرة بالأسبوع	2-4 a week 2-4 بالأسبوع	5-6 a week 5-6 بالأسبوع	1-3 per month 1-3 بالشهر	Less than once per month/ never أقل من مرة بالشهر/ بتاتا
Arabic coffee	القهوة العربي	⬭	⬭	⬭	⬭	⬭	⬭	⬭	⬭	⬭
Other kind of coffee	نوع آخر من القهوة	⬭	⬭	⬭	⬭	⬭	⬭	⬭	⬭	⬭
Black/red tea	الشاي الأسود/ الأحمر	⬭	⬭	⬭	⬭	⬭	⬭	⬭	⬭	⬭
Green tea	الشاي الأخضر	⬭	⬭	⬭	⬭	⬭	⬭	⬭	⬭	⬭
Herbals	الأعشاب (بابونج، يانسون، شمر، حبق، نعناع، زنجبيل، كمون، كركم و غيره)	⬭	⬭	⬭	⬭	⬭	⬭	⬭	⬭	⬭

62. * عدد مرات استهلاك المشروبات باليوم-22 How often do you consume these beverages per day

الأسئلة تعتمد جميعها خلال فترة العزل.

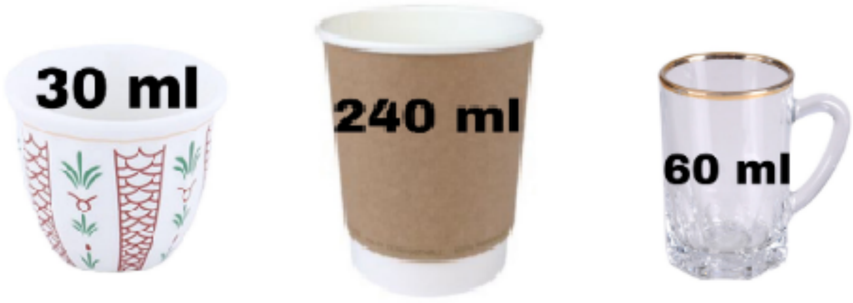

Mark only one oval per row.

		0	1	2	3	4	5 و أكثر
Arabic coffee (Finjan)	عدد فنجال/باليوم - القهوة العربية	⬭	⬭	⬭	⬭	⬭	⬭
Other kind of coffee (cup)	عدد أكواب/باليوم - أنوا ع أخرى من القهوة	⬭	⬭	⬭	⬭	⬭	⬭
Red tea (biallah)	عدد بيالات/باليوم - الشاي الأحمر	⬭	⬭	⬭	⬭	⬭	⬭
Green tea (Biallah)	عدد بيالات/باليوم - الشاي الأخضر	⬭	⬭	⬭	⬭	⬭	⬭
Herbals (Cup)	عدد أكواب/باليوم - الأعشاب (بابونج، يانسون، شمر، حبق، نعنا ع، زنجبيل، كمون، كركم و غيره)	⬭	⬭	⬭	⬭	⬭	⬭

63. * كم تستهلك من المياه يوميا (تقريبا) خلال فترة العزل ؟-23

How often do you consume of water

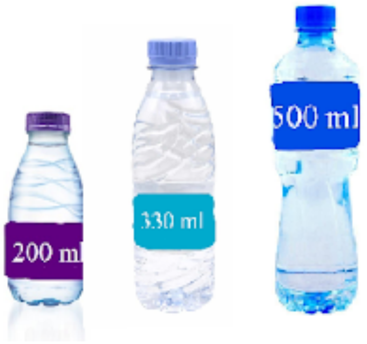

Mark only one oval.

- ⬭ أقل من 500 مل — Less than 500 ml
- ⬭ 1000 مل 1500- مل — 1000-1500 ml
- ⬭ 1600 مل 2000-مل — 1600-2000 ml
- ⬭ أكثر من 2000 مل — More than 2000 ml
- ⬭ اكثر من 2500 مل — More than 2500 ml

64. 24-؟ (سجائر، شيشة، سجائر الكترونية و غيرها من الأنواع) هل أنت مدخن/ة *

Are you smoker?

Mark only one oval.

- نعم Yes
- لا No

Emotional Eating Scale

We all respond to different emotions in different ways. Some types of feelings lead people to experience an urge to eat. Use the form below to identify the extent to which the following feelings lead you to feel an urge to eat, by ticking the appropriate box.

	**No desire to eat**	**A small desire to eat**	**A moderate desire to eat**	**A strong urge to eat**	**An over-whelming urge to eat**
**Resentful**					
**Discouraged**					
**Shaky**					
**Worn out**					
**Inadequate**					
**Excited**					
**Rebellious**					
**Down**					
**Jittery**					
**Sad**					
**Uneasy**					
**Irritated**					
**Jealous**					
**Worried**					

**Frustrated**					
**Lonely**					
**Furious**					
**On edge**					
**Confused**					
**Nervous**					
**Angry**					
**Guilty**					
**Bored**					
**Helpless**					
**Upset**					

Perceived Stress Scale

The questions in this scale ask you about your feelings and thoughts **during the last month**. In each case, you will be asked to indicate by circling *how often* you felt or thought a certain way.

Name ____ Date __________

Age _________ Gender (*Circle*): **M** **F** Other _______________________________________

0 = Never 1 = Almost Never 2 = Sometimes 3 = Fairly Often 4 = Very Often

1. In the last month, how often have you been upset because of something that happened unexpectedly?	**0**	**1**	**2**	**3**	**4**
2. In the last month, how often have you felt that you were unable to control the important things in your life?	**0**	**1**	**2**	**3**	**4**
3. In the last month, how often have you felt nervous and "stressed"?	**0**	**1**	**2**	**3**	**4**
4. In the last month, how often have you felt confident about your ability to handle your personal problems?	**0**	**1**	**2**	**3**	**4**
5. In the last month, how often have you felt that things were going your way?	**0**	**1**	**2**	**3**	**4**
6. In the last month, how often have you found that you could not cope with all the things that you had to do?	**0**	**1**	**2**	**3**	**4**
7. In the last month, how often have you been able to control irritations in your life?	**0**	**1**	**2**	**3**	**4**
8. In the last month, how often have you felt that you were on top of things?	**0**	**1**	**2**	**3**	**4**
9. In the last month, how often have you been angered because of things that were outside of your control?	**0**	**1**	**2**	**3**	**4**
10. In the last month, how often have you felt difficulties were piling up so high that you could not overcome them?	**0**	**1**	**2**	**3**	**4**

Patient Health Questionnaire (PHQ-9)

Patient Name: ______________________________ **Date:** ______________

	Not at all	Several days	More than half the days	Nearly every day
1. Over the *last 2 weeks*, how often have you been bothered by any of the following problems?				
a. Little interest or pleasure in doing things	☐	☐	☐	☐
b. Feeling down, depressed, or hopeless	☐	☐	☐	☐
c. Trouble falling/staying asleep, sleeping too much	☐	☐	☐	☐
d. Feeling tired or having little energy	☐	☐	☐	☐
e. Poor appetite or overeating	☐	☐	☐	☐
f. Feeling bad about yourself or that you are a failure or have let yourself or your family down	☐	☐	☐	☐
g. Trouble concentrating on things, such as reading the newspaper or watching television.	☐	☐	☐	☐
h. Moving or speaking so slowly that other people could have noticed. Or the opposite; being so fidgety or restless that you have been moving around a lot more than usual.	☐	☐	☐	☐
i. Thoughts that you would be better off dead or of hurting yourself in some way.	☐	☐	☐	☐
2. If you checked off any problem on this questionnaire so far, how difficult have these problems made it for you to do your work, take care of things at home, or get along with other people?	Not difficult at all<br>☐	Somewhat difficult<br>☐	Very difficult<br>☐	Extremely difficult<br>☐

Generalized Anxiety Disorder 7-item (GAD-7) scale

Over the last 2 weeks, how often have you been bothered by the following problems?	Not at all sure	Several days	Over half the days	Nearly every day
1. Feeling nervous, anxious, or on edge	0	1	2	3
2. Not being able to stop or control worrying	0	1	2	3
3. Worrying too much about different things	0	1	2	3
4. Trouble relaxing	0	1	2	3
5. Being so restless that it's hard to sit still	0	1	2	3
6. Becoming easily annoyed or irritable	0	1	2	3
7. Feeling afraid as if something awful might happen	0	1	2	3
Add the score for each column	+	+	+	
Total Score *(add your column scores)* =				

s

Name____ Date_________

Sleep Quality Assessment (PSQI)

What is PSQI, and what is it measuring?

The Pittsburgh Sleep Quality Index (PSQI) is an effective instrument used to measure the quality and patterns of sleep in adults. It differentiates "poor" from "good" sleep quality by measuring seven areas (components): subjective sleep quality, sleep latency, sleep duration, habitual sleep efficiency, sleep disturbances, use of sleeping medications, and daytime dysfunction over the last month.

INSTRUCTIONS:

The following questions relate to your usual sleep habits during the past month only. Your answers should indicate the most accurate reply for the majority of days and nights in the past month. Please answer all questions.

During the past month,

1. When have you usually gone to bed? ________________________________
2. How long (in minutes) has it taken you to fall asleep each night? ________________________________
3. What time have you usually gotten up in the morning? ________________________________
4. A. How many hours of actual sleep did you get at night? ________________________________
   B. How many hours were you in bed? ________________________________

5. During the past month, how often have you had trouble sleeping because you	Not during the past month (0)	Less than once a week (1)	Once or twice a week (2)	Three or more times a week (3)
A. Cannot get to sleep within 30 minutes				
B. Wake up in the middle of the night or early morning				
C. Have to get up to use the bathroom				
D. Cannot breathe comfortably				
E. Cough or snore loudly				
F. Feel too cold				
G. Feel too hot				
H. Have bad dreams				
I. Have pain				
J. Other reason (s), please describe, including how often you have had trouble sleeping because of this reason (s):				
6. During the past month, how often have you taken medicine (prescribed or "over the counter") to help you sleep?				
7. During the past month, how often have you had trouble staying awake while driving, eating meals, or engaging in social activity?				
8. During the past month, how much of a problem has it been for you to keep up enthusiasm to get things done?				
9. During the past month, how would you rate your sleep quality overall?	Very good (0)	Fairly good (1)	Fairly bad (2)	Very bad (3)

Physical Activity

Next I am going to ask you about the time you spend doing different types of physical activity in a typical week. Please answer these questions even if you do not consider yourself to be a physically active person.

Think first about the time you spend doing work. Think of work as the things that you have to do such as paid or unpaid work, study/training, household chores, harvesting food/crops, fishing or hunting for food, seeking employment. *[Insert other examples if needed].* In answering the following questions 'vigorous-intensity activities' are activities that require hard physical effort and cause large increases in breathing or heart rate, 'moderate-intensity activities' are activities that require moderate physical effort and cause small increases in breathing or heart rate.

Questions		Response	Code
**Activity at work**			
1	Does your work involve vigorous-intensity activity that causes large increases in breathing or heart rate like *[carrying or lifting heavy loads, digging or construction work*] for at least 10 minutes continuously?<br>*[INSERT EXAMPLES] (USE SHOWCARD)*	Yes 1<br>No 2 *If No, go to P 4*	P1
2	In a typical week, on how many days do you do vigorous-intensity activities as part of your work?	Number of days └─┘	P2
3	How much time do you spend doing vigorous-intensity activities at work on a typical day?	Hours : minutes └─┴─┘ : └─┴─┘ hrs mins	P3 (a-b)
4	Does your work involve moderate-intensity activity that causes small increases in breathing or heart rate such as brisk walking *[or carrying light loads*] for at least 10 minutes continuously?<br>*[INSERT EXAMPLES] (USE SHOWCARD)*	Yes 1<br>No 2 *If No, go to P 7*	P4
5	In a typical week, on how many days do you do moderate-intensity activities as part of your work?	Number of days └─┘	P5
6	How much time do you spend doing moderate-intensity activities at work on a typical day?	Hours : minutes └─┴─┘ : └─┴─┘ hrs mins	P6 (a-b)
**Travel to and from places**			
The next questions exclude the physical activities at work that you have already mentioned.<br>Now I would like to ask you about the usual way you travel to and from places. For example to work, for shopping, to market, to place of worship. [insert other examples if needed]			
7	Do you walk or use a bicycle (*pedal cycle*) for at least 10 minutes continuously to get to and from places?	Yes 1<br>No 2 *If No, go to P 10*	P7
8	In a typical week, on how many days do you walk or bicycle for at least 10 minutes continuously to get to and from places?	Number of days └─┘	P8
9	How much time do you spend walking or bicycling for travel on a typical day?	Hours : minutes └─┴─┘ : └─┴─┘ hrs mins	P9 (a-b)
**Recreational activities**			
The next questions exclude the work and transport activities that you have already mentioned.<br>Now I would like to ask you about sports, fitness and recreational activities (leisure), [insert relevant terms].			
10	Do you do any vigorous-intensity sports, fitness or recreational (*leisure*) activities that cause large increases in breathing or heart rate like [*running or football,]* for at least 10 minutes continuously?<br>*[INSERT EXAMPLES] (USE SHOWCARD)*	Yes 1<br>No 2 *If No, go to P 13*	P10
11	In a typical week, on how many days do you do vigorous-intensity sports, fitness or recreational (*leisure*) activities?	Number of days └─┘	P11
12	How much time do you spend doing vigorous-intensity sports, fitness or recreational activities on a typical day?	Hours : minutes └─┴─┘ : └─┴─┘ hrs mins	P12 (a-b)

Physical Activity (recreational activities) contd.			
**Questions**		**Response**	**Code**
13	Do you do any moderate-intensity sports, fitness or recreational *(leisure)* activities that causes a small increase in breathing or heart rate such as brisk walking,(*cycling, swimming, volleyball*)for at least 10 minutes continuously?<br>*[INSERT EXAMPLES] (USE SHOWCARD)*	Yes 1<br>No 2 *If No, go to P16*	P13
14	In a typical week, on how many days do you do moderate-intensity sports, fitness or recreational (*leisure*) activities?	Number of days └─┘	P14
15	How much time do you spend doing moderate-intensity sports, fitness or recreational (*leisure*) activities on a typical day?	Hours : minutes └─┴─┘ : └─┴─┘<br>hrs mins	P15 (a-b)
**Sedentary behaviour**			
The following question is about sitting or reclining at work, at home, getting to and from places, or with friends including time spent [sitting at a desk, sitting with friends, travelling in car, bus, train, reading, playing cards or watching television], but do not include time spent sleeping.<br>*[INSERT EXAMPLES] (USE SHOWCARD)*			
16	How much time do you usually spend sitting or reclining on a typical day?	Hours : minutes └─┴─┘ : └─┴─┘<br>hrs min s	P16 (a-b)

التعرض للشمس

*الأسئلة تعتمد جميعها خلال فترة العزل.

101. هل تتعرض للشمس بشكل يومي ؟ * Do you expose to the sun daily

Mark only one oval.

- نعم YES
- لا NO

102. إذا كانت الإجابة (نعم) كم دقيقة باليوم؛ و إذا كانت (لا) اكتب (0) *

If yes, specify how many minutes daily

___

103. هل تتعرض للشمس بشكل أسبوعي؟ * Do you expose to the sun weekly

Mark only one oval.

- نعم Yes
- لا No

104. إذا كانت الإجابة (نعم) كم يوم بالأسبوع؛ و إذا كانت (لا) اختار (0) *

Mark only one oval per row. If yes, specify how many days per week

	0	1	2	3	4	5	6	7
عدد الأيام								

105. إذا كانت الإجابة (نعم) كم دقيقة في كل مرة؛ و إذا كانت (لا) اكتب (0) *

If yes, specify how many minutes each time

___

106. ما هو وقت تعرضك للشمس ؟ بإمكانك الخيار أكثر من وقت *

Check all that apply. What it the exposed time? You can choose more the one option

- [ ] عند الشروق On the sunrise
- [ ] عند الظهيرة On the Afternoon
- [ ] قبل الغروب On the Sunset

107. ما هي طبيعة عملك ؟ *

Nature of work

Mark only one oval.

- ( ) داخلي (في الغرف أو المناطق المغطاة) Inside
- ( ) خارجي (تتعرض للشمس بشكل مباشر) Outside

108. ما هي أجزاء الجسم الأكثر تعرضاً للشمس ؟ *

Mark only one oval. What are the most exposed parts of your body?

- ( ) الوجه Face
- ( ) اليدين Hands
- ( ) الوجه واليدين Face and hands
- ( ) الوجه واليدين والقدمين Face, hands and feet

109. ما هو مدى تغطية الملابس للجسم أثناء تعرضك للشمس؟ *

Mark only one oval. How much your clothes cover your body

- ( ) الجسم عدا الوجه واليدين Body except face and hands
- ( ) الجسم عدا الوجه واليدين والرجلين Body except face, hands and feet
- ( ) الجسم عدا الوجه Body except face
- ( ) الجسم بالكامل Whole body
- ( ) الجسم عدا اليدين والرجلين Body except hands and feet

110. هل تستخدم كريمات الحماية من الشمس ؟ *

Mark only one oval. Do you use sunblock?

- ( ) نعم Yes
- ( ) لا No

111. إذا كانت الإجابة (نعم) ما نوع الكريمات الحماية من الشمس ؟ و إذا كانت الإجابة (لا) اكتب (لا يوجد)

If yes, specify the kind of your sunblock

____

شكرا لقد انتهيت من التعبئة أرجو الضغط عل إرسال (submit)

University of Glasgow | Section of Human Nutrition

الهيئة العامة للغذاء والدواء
Saudi Food & Drug Authority

DIET AND NUTRITION
SURVEY IN RIYADH CITY

Date of Interview ☐☐/☐☐/☐☐☐☐ *Participant's Code* ☐☐☐☐

Saudi Food Frequency Questionnaire

1. Dairy Products: الحليب ومنتجاته

During the past year, on average, how often have you consumed the following, dairy foods? (Please check the appropriate box)

Code	Food Item	Per day (times)				Per week			Per month or Never		Measure	Code (office use only)
		6+	4-5	2-3	once	5-6	2-4	once	1-3	Less than once		
**1.1**	**Milk: حليب** Or Flavored											
1.1.1	كامل الدسم Whole											1 cup, 240ml
1.1.2	قليل الدسم Low Fat											
1.1.3	منزوع الدسم Skimmed											
**1.2**	**Laban:لبن**											
1.2.1	كامل الدسم Whole											1 cup, 240ml
1.2.2	قليل الدسم Low Fat											
1.2.3	منزوع الدسم Skimmed											
**1.3**	**Cream Cheese:**(جبنة الكريمة القابلة للدهن(مثل:المراعي،بوك،كرافت											
1.3.1	كامل الدسم Whole											
1.3.2	قليل الدسم Low Fat											
**1.4**	**White cheese (Feta, Mozzarella and Spread Cheeses)** الجبنة البيضاء(فيتا، موزاريلا،الجبنة قابلة للدهن)											1Tbsp, 15g
**1.5**	**Cheddar Cheese** جبنة التشيدر											
**1.6**	**Yogurt:**زبادي/روب (Plain - Flavored with fruits)											
1.6.1	كامل الدسم Whole											1 can, 170g
1.6.2	قليل الدسم Low Fat											
1.6.3	منزوع الدسم Skimmed											
**1.7**	**Labnah:**لبنه											
1.7.1	كامل الدسمWhole											1Tbsp,15g
1.7.2	قليل الدسم Low Fat											1Tbsp,15g
**1.8**	**Ice cream:** آيس كريم Full, low, Skimmed											1 scoop, 33g
**1.9**	**Quashta (Cream) /Gaymar** قشطة/ قيمر											1Tbsp,15g
**1.10**	Yogurt drink شراب الزبادي Type:...........................if applicable											1 can,300ml
**1.11**	Paneer Full, low, Skimmed جبنة بانيير											1Tbsp,15g
**1.12**	Cottage cheese جبنة قريش											1Tbsp,15g
**1.13**	Quarg Full, low, Skimmed											1Tbsp,15g
**1.14**	Sour cream Full, low, Skimmed كريمة حامضة											1Tbsp,15g
**1.15**	Soy Milk حليب الصويا											1Tbsp,15g
**1.16**	Rice Milk حليب الأرز											1 cup,240ml
**1.17**	Curd Full, Low ,Skimmed مجبر											1Tbsp,15g
**1.18**	Cream "Non whipped" Full, Low Skimmed											1 cup,240ml
1.19	Greek Yogurt (plain - flavored) Full, Low, Skimmed											1 can,170g

Majid Alkhalaf

2. <u>Fruits</u>: الفواكه

During the past year, on average, how often have you consumed the following fruits? (please check the appropriate box)

(For seasonal fruits marked *, please estimate your average use when the fruit is in season)

(للفواكه الموسمية ذات العلامة * ، يرجى تقدير متوسط استهلاكك لها في الموسم)

Code	Food Item	Per day (times)				Per week			Per month or Never		Measure	Code (office use only)
		6+	4-5	2-3	once	5-6	2-4	once	1-3	Less than once		
**2.1**	**Fresh Fruit Salad** سلطة فواكه طازجه											1cup,340ml
**2.2**	**Tinned Fruits (all kinds)** فواكه معلبه											1can,425g
**2.3**	**Apple** تفاح											1medium, 180g
**2.4**	**Bananas** موز											1medium, 120g
**2.5**	***Strawberry, raspberries** فراولة، توت											1cup,, 212gm
**2.6**	**Oranges and Mandarin** برتقال ،يوسفي											1 medium, 178g
**2.7**	**Grapefruit** جريب فروت											1 medium, 178g
**2.8**	**Plums, Peaches /Nectarines, or Apricots** برقوق، بخارى،خوخ أو مشمش											2pcs., 70g
**2.9**	**Dates:** تمر											
2.9.1	Dry dates (Tammer) تمر											2pcs, 20g
2.9.2	Fresh Dates (Ruttab, refriger.) تمر مبرد											2pcs,40g
2.9.3	*Fresh (Ruttab or Fresh) رطب أو بلح											2pcs, 20g
**2.10**	**Pears, Pineapple, Kiwi, Figs, Cherries** كمثرى، أناناس، كيوي، تين، كرز											1cup, 240g
**2.11**	**Grapes** عنب											13pcs,90g
**2.12**	**Olives** زيتون											9 medium, 33g
**2.13**	**Dried Fruits (e.g. Figs, Apricots, Raisins, Peaches)** فواكه مجففه(تين،مشمش،زبيب،خوخ)											1 cup, 240g
**2.14**	***Watermelon** بطيخ											1slice,200g
**2.15**	***Other melons (e.g. Canteloupe, Galia or Honeydew)** شمام/بطيخ اصفر											1slice,200g
**2.16**	**Mango** مانجو											1 medium, 300g
**2.17**	**Pomegranate** رمان											1 cup,186g

Please, make sure you have given an answer for every line before leaving this page

University of Glasgow | Section of Human Nutrition

الهيئة العامة للغذاء والدواء
Saudi Food & Drug Authority

3. Vegetables: الخضروات

During the past year, on average, how often have you consumed the following vegetables? (please check the appropriate box)

Code	Food Item	Per day (times)				Per week			Per month or Never		Measure	Code (office use only)
		6 +	4-5	2-3	once	5-6	2-4	once	1-3	Less than once		
3.1	**Cabbage:** ملفوف/كرنب											
3.1.1	طازج Fresh											1cup,100g
3.1.2	مطبوخ Cooked											1 cup,252g
3.2	**Mixed Vegetable Salad (fresh)** سلطة مشكلة											1 cup,234g
3.3	**Mixed Vegetables (cooked)** خضار مشكلة مطبوخة											
3.4	**Cauliflower, Broccoli or Brussels sprouts (cooked)** زهرة،القرنبيط المطبوخ أو كرنب											1 cup, 294g
3.5	**Tabouleh** تبولة											1cup,234g
3.6	**Green leafy vegetables** (Molokhia & spinach) الورقيات الخضراء (ملوخية، سبانخ), **Okra & Green Beans** باميا، لوبا فاصوليا خضراء											1cup, 100g
3.7	**Peas (cooked)/ (bazelah)** بازلاء مطبوخة											1cup,240g
3.8	**Carrots** جزر											1 cup, 174g
3.9	**Tomatoes** طماطم											1 cup, 290g
3.10	**Mushrooms** فطر(مشروم)											1 cup, 254g
3.11	**Leeks** كراث											1 cup, 72g
3.12	**Cucumber** خيار											1 cup, 194g
3.13	**Lettuce** خس											1 cup, 66g
3.15	**Sweetcorn** ذرة											1 can, 340g
3.16	**Onion** بصل											1 cup, 200g
3.17	**Beans (fava, red, white and Soya beans), lentils, Pigeon, Chick & cow peas) (cooked)** فول،عدس،فول الصويا،فاصوليا،حمص(بليلة) فاصوليا											1 cup,220g
3.18	**Baked beans** فاصوليا حلوة											1 cup, 312g
3.19	**Boiled potatoes** بطاطس مطبوخة (مسلوقه)											1 small,96g
3.20	**Fried potatoes (French fries)**(اصابع) بطاطس مقلية											1 cup,80g
3.21	**Potato crisps** بطاطس تشيبس (مثل برينقلز أو ليز)											1 small pack 26g

Please, make sure you have given an answer for every line before leaving this page

Majid Alkhalaf

University of Glasgow | Section of Human Nutrition

الهيئة العامة للغذاء والدواء
Saudi Food & Drug Authority

4. Meat, Fish and Eggs: لحوم، أسماك وبيض

During the past year, on average, how often have you consumed the following meat, fish, eggs etc.? (please check the appropriate box)

Code	Food Item	Per day (times)				Per week			Per month or Never		Measure	Code (office use only)
		6+	4-5	2-3	once	5-6	2-4	once	1-3	Less than once		
4.1	**Boiled Egg** بيض مسلوق											1 medium, 60g
4.2	**Fried eggs, Scrambled eggs or omelets** بيض مقلي، أمليت											1 medium, 60g
4.3	**Chicken or turkey (boiled, grilled or fried)** دجاج، رومي(مسلوق،مشوي،مقلي)											60 g
4.4	**Lamb or mutton as a mixed dish (e.g. Kabsah, stew, casserole, Spaghetti, lasagne etc.** لحم ضأن مضاف لأطباق مشكله كبسة لحم,.ايدام/مرق، سباغيتي ولازانيا باللحم											100g
4.5	**Lamb or mutton as a main dish (eg. Steak, roast, in gravy etc.)** لحم ضأن طبق أساسي، ستيك، اضلاع مشوي أو في مرق/ايدام											180g
4.6	**Beef as a or mixed dish (e.g. Kabsah, stew, casserole, Spaghetti, lasagne etc.** لحم بقري في طبق متنوع من الحوم البقرية (كبسة،يدام/ مرق، سباغتي، لازانيا)											100g
4.7	**Beef as a main dish (eg. Steak, roast, in gravy etc.)** لحم بقري لطبق اساسي ستيك، اضلاع مشوي أو في ايدام/مرق											180g
4.8	**Camel Meat** لحم جمل											100g
4.9	**Burgers (beef burger or Chicken Burger)** برجر (لحم بقري او دجاج)											90g
4.10	**Fried fish** سمك مقلي											60g
4.11	**Grilled or steamed fish** سمك مشوي أو سمك بالبخار											60g
4.12	**Canned tuna, salmon, mackerel, or sardine** تونة معلبه سلمون، الإسقري، سردين											183g
4.13	**Prawn or Shrimps** جمبري او ربيان											1pcs., 18g
4.14	**Mussels, oysters, scallops or cockles** بلح البحر، المحار، اسكلوب، الصدفيات											85g
4.15	**Organ meat (liver,brain,heart)** Kidney لحوم الاحشاء(كبد، مخ،قلب)											100g
4.16	Goat لحم الماعز											100g
4.17	White fish سمك أبيض											100g
4.18	Tofu											100g
4.19	Pork											

Please, make sure you have given an answer for every line before leaving this page

Majid Alkhalaf

5. Mixed Dishes: أطباق مختلطة

During the past year, on average, how often have you consumed the following mixed dishes? (please check the appropriate box)

Code	Food Item	Per day (times)				Per week			Per month or Never		Measure	Code (office use only)
		6+	4-5	2-3	once	5-6	2-4	once	1-3	Less than once		
5.1	**Vegetable soup** شوربة خضار											
5.2	**Chicken soup with cream** شوربة الدجاج بالكريمه											1 cup, 240ml
5.3	**Lentil soup** شوربة عدس											
5.4	**Oat meal Soup** شوربة الشوفان											
5.5	**Jareesh (gerish), Marqooq, harees, mataziz, or Qorsan** جريش،مرقوق،هريس،مطاطيز،قرصان											1/2plate, 340g
5.6	**Saleeq** سليق/رز طايفي											
5.7	**Marasia (Marassia)** مراصيع											1/2plate, 60g
5.8	**Aseeda (Asseda)** عصيد											1/2plate,340g
5.9	**Rice (Kabsah, Beriani, Bukhari or mandi) without meat** أرز(كبسة،برياني،بخاري،مندي)بدون لحم											1/2 plate, 180g
5.10	**Rice with Vegetables (Maqloobah) without meat** أرز بالخضار(مسلوق) بدون لحم											1/2 plate, 180g
5.11	**Kebbah** كبه											1/2plate,288g
5.12	**Mottabaque W/ egg (Muttabagh)** مطبق بالبيض											1/2 plate, 120g
5.13	**Stuffed vegetables (Vine leaves, cabbage, squash)** خضراوات محشية (ورق عنب،ملفوف،كوسا)											1 finger, 15g
5.14	**Eggplant (aubergine), Zucchini (Marrow) (cooked) (Mussakaah OR Moussaka)** باذنجان،كوسة(مصقعه)											1/2 plate, 354g
5.15	**Lasagne** لازانيا											1/2 plate, 470g

Code	Food Item	6+	4-5	2-3	once	5-6	2-4	once	1-3	Less than once	Measure	Code (office use only)
5.16	Marmite/ مستخرج الخطيرة القابل للدهن kraft vegetarian vegemite											1 Tbsp, 15g
5.17	Vegetarian meat substitutes (e.g.vegetarian sausage, patties). بدائل اللحم للنباتيين											

Please, make sure you have given an answer for every line before leaving this page

6. Sandwiches and Snacks: سندويشات ووجبات خفيفة

During the past year, on average, how often have you consumed the following sandwiches and snacks? (please check the appropriate box)

Code	Food Item	Per day (times)				Per week			Per month or Never		Measure	Code (office use only)
		6+	4-5	2-3	once	5-6	2-4	once	1-3	Less than once		
6.1	**Belila / chick peas** بليله/ الحمص											1 cup, 270g
6.2	**Falafel** فلافل											1 medium, 42g
6.3	**Shawerma (Doner Kebab)** شاورما											1 small, 111g
6.4	**Sambosa with meat or cheeses** سمبوسة بالحم او الجبن											1 pcs., 21g
6.5	**Fatayer (pies) with cheese, meat and vegetables** فطائر بالجبن اوالحم او الخضار											1 pcs., 30g
6.6	**Pizza (all)** بيتزا											1 slice, 30g
6.7	**Meat Sandwich (chicken or meat)** ساندويتش لحم											1 small,111g
6.8	**Cheese sandwich** ساندويتش بالجبن											1 samoli, 90g
6.9	**Egg sandwich (omelette,shakshokah or boiled)** ساندويتش بيض(اومليت، شكشوكة ،مسلوق)											1 samoli, 120g
6.10	**Offal sandwich (liver or kidney)** ساندويتش كبده أو كلاوي											1 small, 111g
6.11	**Jam sandwich** ساندويتش مربى											1 samoli, 90g
6.12	**Hamoose (paste)** حمص											1 Tbsp, 15g

7. Breads, Cereals and Starches: مخبوزات، حبوب ونشويات

During the past year, on average, how often have you consumed the following Breads, cereals and starches? (Please check the appropriate box)

Code	Food Item	Per day (times)				Per week			Per month or Never		Measure	Code (office use only)
		6 +	4-5	2-3	once	5-6	2-4	once	1-3	Less than once		
7.1	**Breakfast cereals (corn flakes)** رقائق الذرة (كورن فلكس)											1cup, 40g
7.2	**White bread (Samoli, tabonah, tannor, toast, pita)** خبز ابيض(صامولي،تنور،توست											1/2 samoli, 1 slice toast, 30 g
7.3	**Brown bread (Samoli, Tabonah, toast, pita)** خبز بر (صامولي، توست)											1/2 samoli, 1 slice toast, 30 g
7.4	**Tamees or Irani bread with sesame** تميس او خبز ايراني مع السمسم											1 slice, 70g
7.5	**Pasta spaghetti and Macaroni** مكرونه(باستا،سباغتي)											1 cup, 124g
7.6	**Dates Biscuits** بسكوت بالتمر											1 pcs, 33g
7.7	**Other Biscuits and crackers** كل أنواع البسكويت											1pcs,.9g
7.8	رقائق الإفطار مع المكسرات Breakfast cereals with nuts											1cup, 40g
7.9	Bran Flakes رقائق النخالة											1cup, 40g
7.10	Rusks شابورة										*Majid Alkhalaf*	1 slice, 13g
7.11	Granola (Plain, Fruits, Nuts)											1cup, 40g

8. Beverages, Juices and Drinks:العصائر والمشروبات

During the past year, on average, how often have you consumed the following beverages?

Code	Food Item	Per day (times)				Per week			Per month or Never		Measure	Code (office use only)
		6+	4-5	2-3	once	5-6	2-4	once	1-3	Less than once		
8.1	**Regular Coffee and** Instant coffee **(e.g. Nescafe)** القهوة السوداء والتركية مثل النسكافيه											1Tbsp, 15gm
8.2	**Arabic Coffee** قهوة عربية											1 finjal, 30ml
8.3	**Coffee Whitnesrs e.g. coffee-mate**(مبيض القهوة (كوفي ميت											1Tbsp, 15gm
8.4	**Regular fizzy soft drink (e.g. Cola, lemonade, etc.)** مشروبات غازية											1 can,330ml
8.5	**Low calorie (diet) fizzy soft drink (e.g. Cola, lemonade, etc.)** مشروبات غازية (بدون سكر)											1 can,330ml
8.6	**Black Tea** شاي اسود											1 bialah, 60ml
8.7	**Green Tea** شاي اخضر											1 bialah, 60ml
8.8	**Fruit squash or cordial** المشروبات المضاف لها ماء (تانج أو فيمتو)											1Tbsp,15gm
8.9	**Hot chocolate/ Cocoa drinks** هوت تشوكليت											1 cup, 240ml
8.10	**Horlicks/Ovaltine/ Nesquik/ Milo** بودرة معززة الطعم تضاف للحليب أو الماء الساخن (نسكويك أو مايلو)											1Tbsp, 15gm
8.11	**Mineral water (not in other drinks) (sehha)** مياه الشرب المعبأة أو المياه المعدنية											330 ml or 600 ml
8.12	**Tap water (not in other drinks)** ماء الصنبور أو الحنفية											1 cup, 240ml
8.13	**Pure fruit juice (orange, apple,etc.)** عصير الفاكهه (برتقال، تفاح أو غيرها)											1 cup, 240ml
8.14	**Energy Drinks** مشروبات الطاقة											1 can,250ml

Brands: V energy drink, Red bull , Powerade

Demon. Other Specify:........................

8.15	**Powdered Milk** حليب بودرة Full, Low, Semi Skimmed											1Tbsp, 15gm

Please, make sure you have given an answer for every line before leaving this page

Majid Alkhalaf

9. Sweets: الحلويات

During the past year, on average, how often have you consumed the following sweet baked goods? (please check the appropriate box)

Code	Food Item	Per day (times)				Per week			Per month or Never		Measure	Code (office use only)
		6+	4-5	2-3	once	5-6	2-4	once	1-3	Less than once		
9.1	Cream caramel كريم كراميل											1cup, 324g
9.2	Halawah Tahinah (Tahini)طحينية											100g
9.3	Doughnuts دونات											1pcs., 60g
9.4	Basbousah and Konafah بسبوسة وكنافه											1/2 plate, 124g
9.5	Mohallabiah مهلبية											100g
9.6	Klijah & Lugimat Al-Ghadi (Lugimat) كليجة و لقيمات											34g
9.7	Cake (All) كيك											100g
9.8	Arabic sweets Baklawa حلويات عربية (بقلاوة)											100g
9.9	Chocolate شكولاته											45g
9.10	Honeyعسل											1Tbsp,15g
9.11	Jam مربى											1Tbsp,15g
9.12	Jellies, All Kinds جلى											1cup, 324g
9.13	Sweet Muffins											60g
9.14	Cheese Muffins											60g
9.15	Cheese Scones											

10. Seeds and Nuts: المكسرات والبذور

During the past year, on average, how often have you consumed the following seeds? (please check the appropriate box)

Code	Food Item	Per day (times)				Per week			Per month or Never		Measure	Code (office use only)
		6 +	4-5	2-3	once	5-6	2-4	once	1-3	Less than once		
10.1	Almondاللوز											1cup,150g
10.2	Cashew nuts كاجو/سن العجوز											1cup,150g
10.3	Peanut فول سوداني											1cup,150g
10.4	Pistachio فستق											1cup,110g
10.5	Walnutجوز											1cup,150g
10.6	Hazelnuts بندق											1cup,150g
10.7	Pecan nut عين الجمل											1cup,150g
10.8	Chest nut أبو فروه											1cup,150g
10.9	Mixed nuts مكسرات مشكله											1cup,188g
10.10	Seeds (sunflower, melon, sesame seeds) بذور الحبحب، القرع أو دوّار الشمس											1cup,110g
10.11	Peanut butterزبدة الفول السوداني											1Tbsp,15g

Please, make sure you have given an answer for every line before leaving this page

11.How often do you eat meals at a fast food/non-fast food restaurant?

في الغالب، ما هو معدل استهلاكك للوجبات الغذائية خارج المنزل سواء من مطاعم الوجبات السريعة أو غيرها ؟

Average Frequency										
Per day (times)				Per week			Per month			Office use only
6+	4-5	2-3	once	5-6	2-4	once	1-3	Never, less than once/ month		
☐	☐	☐	☐	☐	☐	☐	☐	☐		

12.Artificial Sweeteners: المحليات الاصطناعية

Do you take Artificial Sweeteners regularly? هل تستخدم المحليات الاصطناعية ؟

☐ No لا ☐ Yes نعم

If Yes, what kind (including brand name) and how often do you consume.

إذا كانت الإجابة بنعم، ما هو نوع هذا المحلي الاصطناعي (بما في ذلك الاسم/العلامة التجارية) وكم الكمية المستهلكة في الغالب؟

Artificial Sweetener		Average Frequency									
Name &Brand	Dose	Per day (times)				Per week			Per month		Code (office use only)
		6+	4-5	2-3	once	5-6	2-4	once	1-3	Never, less than once/ month	

13.Vitamins and Minerals: الفيتامينات والمعادن

Do you take supplements such as vitamins or minerals regularly?

هل تأخذ المكملات الغذائية مثل الفيتامينات أو المعادن بانتظام؟

☐ No لا ☐ Yes نعم

If Yes, what kind (including brand name) and how often do you consume.

-Formula / Protein supplements / Protein Bars
-Multivitamin
-Vit.D
-Folic Acid
-Iron
-زيت كبد الحوت

إذا كانت الإجابة بنعم، ما هو نوع المكمل الغذائي (بما في ذلك الاسم/العلامة التجارية) وكم الكمية المستهلكة في الغالب؟

Supplement		Average Frequency									
Name &Brand	Dose	Per day (times)				Per week			Per month		Code (office use only)
		6+	4-5	2-3	once	5-6	2-4	once	1-3	Never, less than once/ month	

هل تأخذين أي أعشاب ؟ (يانسون ، شمر ، حبق، نعناع ، زنجبيل ....) نعم لا

ماهو نوعه؟...............

منذ متى تتناولينها؟.............

هل تتناولينها بإنتظام؟ نعم لا

Majid Alkhalaf

كم مرة؟. يوميا. ٣-٤ في الأسبوع. مرة في الأسبوع الكمية؟.......................

14. Fats, Oils and Sugar: الدهون، الزيوت والسكر

A. Spreads and Oils: (الدهون والزيوت القابلة للدهن، عدا التي تستخدم في الطبخ)

Do you use any butter, margarine or other spread or oil on bread?
هل تستخدم الزبدة، السمن، المارجرين أو الزيوت على الخبز؟

☐ No لا ☐ Yes نعم

If yes, please give full details of the type(s) you use most often?
إذا كانت الإجابة بنعم، يرجى إعطاء التفاصيل الكاملة للأنواع التي تستخدم في معظم الأحيان؟

Office code

……………………………………………………………………………………. ☐☐

……………………………………………………………………………………. ☐☐

……………………………………………………………………………………. ☐☐

……………………………………………………………………………………. ☐☐

If yes, how many teaspoons of butter, margarine or other spread did you use each day on bread?
إذا كان الجواب نعم، كم ملعقة شاي تستخدم يومياً من الزبدة، المرجرين، السمن أو الزيوت الأخرى التي استخدمتها على الخبز؟

☐☐

B. Sugar: السُكّر

Do you add sugar on your tea or coffee? هل تقوم بإضافة السكر على الشاي، القهوة، الكورن فليكس أو غيرها الخاصة بك؟

☐ No لا ☐ Yes نعم

If yes, what type of sugar you are using? إذا كان الجواب نعم، فما هو نوع السكر المستخدم

☐ White سُكّر أبيض ☐ Brown سُكّر بُنّي

☐ Others (غيرها) ……………………………………………………………… (include brand names please)

(بما في ذلك الاسم أو العلامة التجارية)

How many teaspoons of table sugar did you use each day in drinks and on cereals or deserts?
إذا كان الجواب نعم، كم من ملعقة شاي من سكر المائدة التي تستخدمها كل يوم في المشروبات، الكورن فليكس وغيرها

☐☐

(If you did not use any table sugar, please enter 0). (إذا كنت لا تستخدم أي كمية من سكر المائدة، من فضلك ضع (0)).

C. Salt: ملح الطعام

Do you add Salt to food while cooking? هل تقوم بإضافة الملح إلى طعامك أثناء الطبخ؟

☐ Never لا ☐ Rarely نادراً ☐ Sometimes أحياناً

☐ Usually عادة ☐ Always دائماً

Please, make sure you have given an answer for every line before leaving this page

Do you add Salt to any food at the table (while eating)? هل تقوم بإضافة الملح إلى طعامك أثناء الأكل؟

☐ **Never** لا ☐ **Rarely** نادراً ☐ **Sometimes** أحياناً

☐ **Usually** عادة ☐ **Always** دائما

Do you regularly use a salt substitute (e.g. LoSalt)? هل تستخدم بدائل الملح بانتظام (على سبيل المثال: لوسولت)؟

☐ **No** لا ☐ **Yes** نعم

If yes, which brand? ....

D. Cooking fats and oils: دهون وزيوت الطهي/الطبخ

Do you use any fat or oil for home frying or cooking? هل تستخدم أي دهون أو زيت للقلي أو الطبخ في المنزل؟

☐ **No** لا ☐ **Yes** نعم

If yes, what type of cooking oil is usually used at home and what is the frequency of consumption?

إذا كانت الإجابة بنعم، ما هو نوع زيت الطهي/الطبخ المستخدم عادة في المنزل وما هو معدل الاستهلاك؟

Code	Food Item	Per day (times)				Per week			Per month or Never		Measure	Code (office use only)
		6+	4-5	2-3	once	5-6	2-4	once	1-3	Less than once		
15.1	**Butter** زبدة											1 Tbsp,15g
15.2	**Margarine** (مارجارين) زبدة نباتية											1 Tbsp,15g
15.3	**Animal Fat (Tallow or Dripping)** شحم حيواني											1 Tbsp,15g
15.4	**Vegetable Oils (Represent. for Sunflower, Canola, Corn, Cottonseed, Palm and Soybean Oils)** زيوت نباتية											1 Tbsp,15g
15.5	**Olive oil** زيت زيتون											1 Tbsp,15g
15.6	**Ghee (represent. palm and butter)** سمن نباتي أو حيواني											1 Tbsp,15g
15.7	**Mayonnaise** مايونيز											1 Tbsp,15g
